# Targeting hemoglobin receptors IsdH and IsdB of *Staphylococcus aureus* with a single VHH antibody inhibits bacterial growth

**DOI:** 10.1016/j.jbc.2023.104927

**Published:** 2023-06-15

**Authors:** Sandra Valenciano-Bellido, Jose M.M. Caaveiro, Makoto Nakakido, Daisuke Kuroda, Chihiro Aikawa, Ichiro Nakagawa, Kouhei Tsumoto

**Affiliations:** 1 Department of Bioengineering, School of Engineering, The University of Tokyo, Tokyo, Japan; 2 Laboratory of Global Healthcare, Graduate School of Pharmaceutical Sciences, Kyushu University, Fukuoka, Japan; 3 Department of Chemistry and Biotechnology, Graduate School of Engineering, The University of Tokyo, Tokyo, Japan; 4 Research Center for Drug and Vaccine Development, National Institute of Infectious Diseases, Tokyo, Japan; 5 Department of Microbiology, Graduate School of Medicine, Kyoto University, Kyoto, Japan; 6 Institute of Medical Science, The University of Tokyo, Tokyo, Japan

**Keywords:** *Staphylococcus aureus*, hemoglobin, heme, iron surface determinant system, IsdH, IsdB, single-domain antibody, alanine scanning, antibiotic action, X-ray crystallography

## Abstract

Methicillin-resistant *Staphylococcus aureus*, or MRSA, is one of the major causative agents of hospital-acquired infections worldwide. Novel antimicrobial strategies efficient against antibiotic-resistant strains are necessary and not only against *S. aureus*. Among those, strategies that aim at blocking or dismantling proteins involved in the acquisition of essential nutrients, helping the bacteria to colonize the host, are intensively studied. A major route for *S. aureus* to acquire iron from the host organism is the Isd (iron surface determinant) system. In particular, the hemoglobin receptors IsdH and IsdB located on the surface of the bacterium are necessary to acquire the heme moiety containing iron, making them a plausible antibacterial target. Herein, we obtained an antibody of camelid origin that blocked heme acquisition. We determined that the antibody recognized the heme-binding pocket of both IsdH and IsdB with nanomolar order affinity through its second and third complementary-determining regions. The mechanism explaining the inhibition of acquisition of heme *in vitro* could be described as a competitive process in which the complementary-determining region 3 from the antibody blocked the acquisition of heme by the bacterial receptor. Moreover, this antibody markedly reduced the growth of three different pathogenic strains of MRSA. Collectively, our results highlight a mechanism for inhibiting nutrient uptake as an antibacterial strategy against MRSA.

The methicillin-resistant strains of *Staphylococcus aureus* (MRSA) are one of the leading causes of morbidity and mortality related to hospital-acquired infections worldwide (1, 2, 3, 4). There is thus a need to develop new antibacterial strategies. *S. aureus* have to survive in a nutrient-deprived environment, with continuous attacks from the immune system of the host defense system, and from the action of antimicrobial compounds such as β-lactam antibiotics (5). Most pathogens, including *S. aureus*, are equipped with different virulence factors (2, 4) as well as antibiotic resistance factors enhancing their survival in an environment with limited nutrients and directed against the immune defenses of the host.

The essential nutrient iron functions as a cofactor in many essential metabolic processes, and therefore, it is important for the growth of pathogenic bacteria such as *S. aureus*. Indeed, several virulence factors have been identified to participate in the acquisition of iron, such as Shr, FtsB IsdX, or yersiniabactin facilitating the growth of the bacteria in the otherwise hostile environment of the host (6, 7, 8, 9, 10). Bacteria have to extract iron from iron- or heme-containing proteins from the host, such as hemoglobin (Hb) (11), to survive and induce infection. Under such conditions, iron restriction would be one of the factors limiting the growth of *S. aureus*. The capture of heme from the host’s heme-carrying proteins, such as Hb, is a challenge and an opportunity for bacterial pathogens (12). The best characterized gram-positive heme transport system is that of *S. aureus* (13). Heme acquisition by *S. aureus* is carried out by a group of proteins collectively termed iron surface determinant (Isd) system. The two surface proteins anchored in the peptidoglycan cell wall, IsdH and IsdB, directly extract heme from Hb, Met-Hb, and Hb–haptoglobin complexes (14, 15, 16). Heme is subsequently transported from the surface proteins IsdH and IsdB across the cell wall and through the plasma membrane by proteins IsdA, IsdC, IsdE, and IsdF. Upon reaching the cytoplasm, heme is degraded by IsdG and IsdI so that the iron atom is released for use in various cellular processes (15, 16, 17). The function of IsdD, also present in the Isd operon, is still unknown.

The Isd system, and in particular surface proteins IsdH and IsdB, is considered to be necessary for the acquisition of the iron atom from heme and for the survival and infectivity of *S. aureus* (18). IsdH and IsdB comprise three and two NEAr-iron transport (NEAT) domains, respectively, and these NEAT domains are connected by linker regions in each protein. It has been reported that the NEAT domain is present in other species of microorganisms; however, the proteins of the Isd system are exclusively expressed in *Staphylococcus* species. IsdH and IsdB are therefore a promising and specific target to develop new antibacterial strategies against MRSA. The three NEAT domains of IsdH are connected by linker regions. NEAT1, NEAT2, and the linker regions of IsdH bind and destabilize the heme-binding pocket of Hb, thus facilitating the extraction of heme by the NEAT3 domain (14, 17). IsdB contains one fewer NEAT domain, and in this protein, the NEAT1 and linker domains are sufficient to recognize and destabilize Hb, resulting in heme acquisition by the NEAT2 domain.

We sought to obtain an antibody capable of impairing the biological activity of IsdH and IsdB by blocking heme binding to them. To that end, we employed the variable domain of heavy chain of heavy chain–only antibody (VHH) obtained from camelid immunoglobins. VHH (also termed nanobody) is specialized in recognizing hidden and hydrophobic epitopes (19) such as that of the heme-binding pocket of IsdH and IsdB. In addition, VHH has other advantages over the traditional IgG format, such as a lower cost of production and, because of their smaller size, greater penetration in targets with difficult accessibility (14, 20).

Using IsdH linker-NEAT3 as the antigen to immunize alpaca, suitable VHHs were obtained by phage display and selected by ELISA. The binding of selected VHH candidates to the antigen was characterized by several high-resolution biophysical techniques such as surface plasmon resonance (SPR) and isothermal titration calorimetry (ITC). The selected VHH candidate recognized IsdH linker-NEAT3 with high affinity. The same VHH also interacted with IsdB linker-NEAT2 with high affinity. X-ray crystallography and molecular dynamics (MD) simulations were employed to reveal the binding mechanism from a structural viewpoint, and the conclusions were strengthened by alanine scanning. Although the VHH did not impair the binding of human Hb (hHb) to IsdH, it blocked heme transfer to the NEAT3 domain. Nearly identical conclusions were obtained when IsdB was employed. Importantly, the growth of three different MRSA strains of *S. aureus* under iron-limiting conditions and in the presence of VHH was largely and consistently reduced.

## Results

### Preparation of VHHs binding to IsdH linker-NEAT3

**Figure 1:**
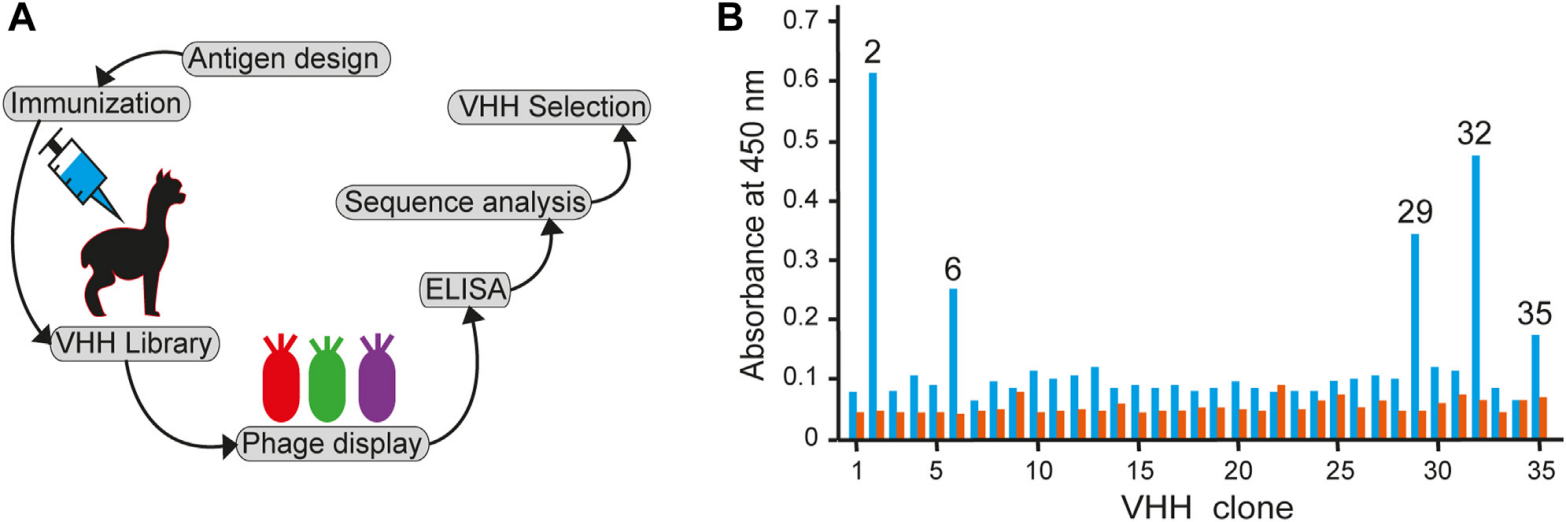
**Preparation of anti-IsdH linker-NEAT3 VHH antibody.***A*, overview. *B*, ELISA. The *blue bar* corresponds to the absorbance of samples each of every different VHHs and the antigen IsdH linker-NEAT3, whereas the *orange bar* corresponds to the absorbance of the control experiment without antigen present. Only antibodies with absorbance significantly greater than their corresponding control were selected for sequencing and analysis. The antibodies that were selected for further analysis are indicated by their identification number at the *top* of their absorbance bar. The sequences of the selected antibodies are given in Table S1. NEAT, NEAr-iron transporter; VHH, variable domain of heavy chain of heavy chain–only antibody.

After immunization of a single individual alpaca with purified IsdH linker-NEAT3 (heme-free form), and following standard procedures, we advanced to obtain a phage library as described in the Experimental procedures section. Phage display (21) with two rounds of acid elution was performed to limit the selection only to antibodies with high affinity. After rounds of selection, a screening of high-affinity VHHs was performed by ELISA under qualitative conditions (Fig. 1*A*). Of the 36 antibodies analyzed, five highly specific VHHs were selected based on the increase of fluorescence intensity with respect to most other samples (Fig. 1*B*). The sequence of the antibodies revealed that the only differences in all five VHHs were limited to the first two residues of the N-terminal region (Table S1); the rest of the sequence was identical, including the hypervariable complementary-determining regions (CDRs). The VHHs could be divided in three groups based on their sequence identity. First, VHH2 and VHH6 were identical to each other and displayed a Glu-Leu at the N-terminal region. Second, VHH29 and VHH32 were also identical to each other and exhibited a Glu-Val sequence at the N-terminal region. And third, VHH35 started with an N-terminal Gln-Leu (Table S1). Although sequence differences outside the CDR generally do not lead to significant differences in affinity (22, 23), we selected two VHHs for in-depth characterization and analysis maximizing the differences in the sequence. VHH6 (bearing a negatively charged residue and a hydrophobic residue at the N terminus) and VHH35 (displaying a polar and a hydrophobic residue) were selected.

### Binding of VHH to IsdH

**Figure 2:**
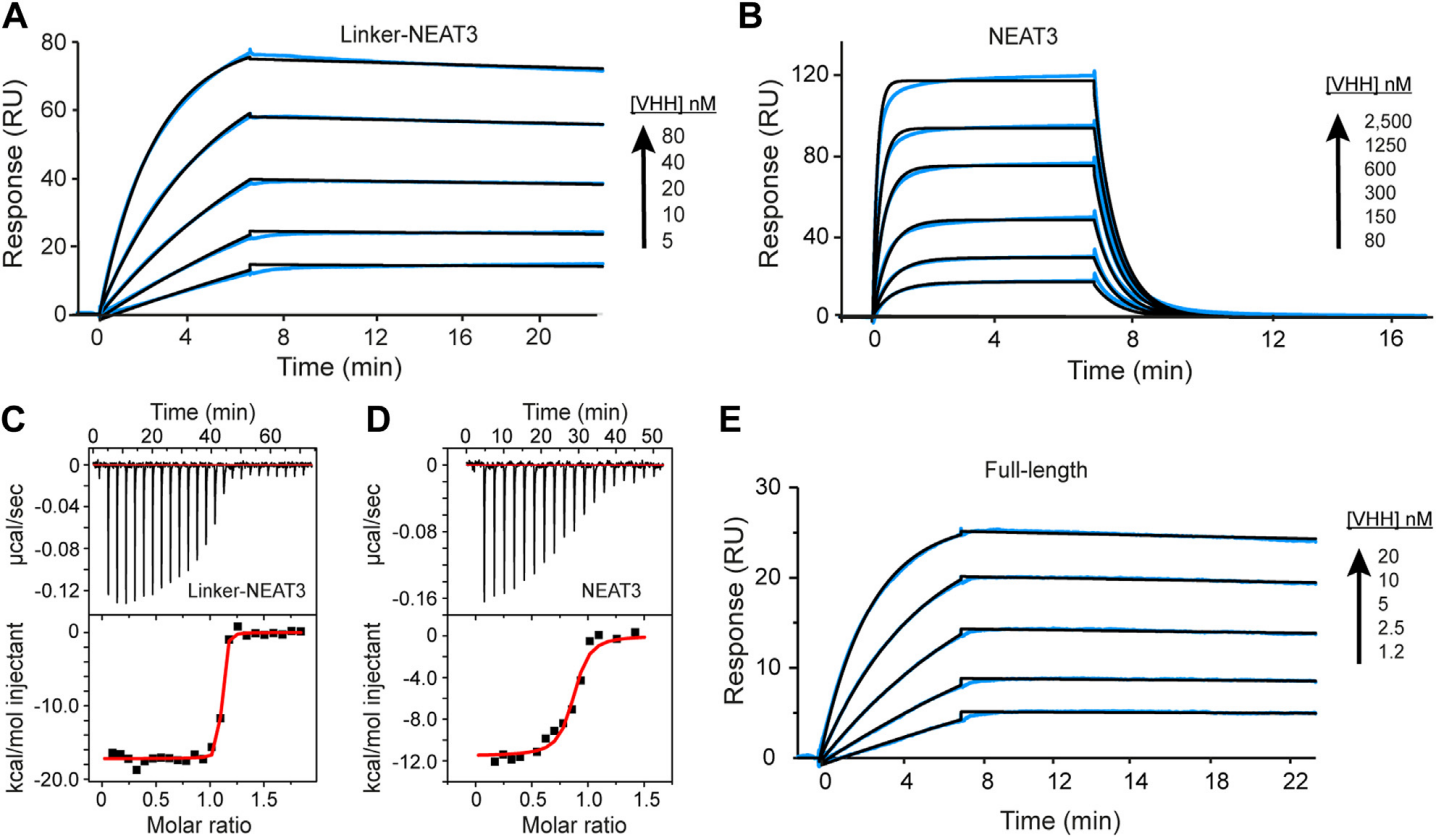
**Binding of VHH6 to IsdH.***A*, SPR and (*B*) ITC data corresponding to the binding of VHH6 to IsdH linker-NEAT3. *C*, SPR and (*D*) ITC data corresponding to the binding of VHH6 to IsdH NEAT3. *E*, binding of VHH6 to IsdH NEAT1–NEAT3 (full-length IsdH). The kinetic and thermodynamic parameters are given in Table 1. Shown are representative data of independent duplicates. ITC, isothermal titration calorimetry; NEAT, NEAr-iron transporter; SPR, surface plasmon resonance; VHH, variable domain of heavy chain of heavy chain–only antibody.

We employed SPR and ITC for detailed characterization of the binding of VHH6 and VHH35 to the antigen IsdH. In this case, we also selected two antigens with increasing size: IsdH NEAT3 and IsdH linker-NEAT3. For the SPR experiment, the antigens were immobilized by the method of amine coupling, and the time course of SPR signal after each VHH was flowed at various protein concentrations (1.2 nM–2.5 μM) monitored and analyzed with the BIAevaluation software (Cytiva) (Table 1; Figs. 2 and S1). When immobilizing IsdH linker-NEAT3, the value of the dissociation constant (*K*_*D*_) was identical for both antibodies (0.53 nM). The values of the individual association (*k*_on_) and dissociation (*k*_off_) rates were also comparable: The values of *k*_on_ and *k*_off_ for VHH6 were 8.1 × 10^−4^ M^−1^ s^−1^ and 0.42 × 10^−4^ s^−1^ and for VHH35 6.6 × 10^−4^ M^−1^ s^−1^ and 0.35 × 10^−4^ s^−1^, respectively. When the immobilized antigen was the NEAT3 domain (no linker), a dramatic increase in the rate of dissociation *k*_off_ was observed, whereas only a modest decrease in *k*_on_ occurred. Specifically, VHH6 dissociated from NEAT3 approximately 560 times faster than from linker-NEAT3, resulting in an approximately 1200-fold lower affinity for the antigen lacking the linker domain. A decrease of affinity was also determined for the binding of VHH35 to NEAT3 without linker (450-fold lower). We also performed an experiment with full-length IsdH immobilized, and in this case, the affinity improved to 0.09 nM, which is approximately six times stronger than that of IsdH linker-NEAT3. This result indicates that NEAT1 and NEAT2 contribute, albeit modestly, to the recognition by VHH6 (Table 1 and Fig. 2*E*). These data clearly indicate that both VHHs recognized the linker-NEAT3 region of IsdH and also that both linker and NEAT3 domains significantly contributed to the high-affinity binding to the antibody.

**Table 1:** Binding of VHHs to various constructs of IsdH^a^

Protein	SPR			ITC	
	*k*_on_ (×10^4^ M^−1^ s^−1^)	*k*_off_ (×10^−4^ s^−1^)	*K*_D_ (nM)	n (mol:mol)	*ΔH* (kcal mol^−1^)
Linker-NEAT3 + VHH6	8.1 ± 0.1	0.42 ± 0.02	0.53 ± 0.02	1.10 ± 0.03	−17.1 ± 0.2
NEAT3 + VHH6	3.7 ± 0.2	235.2 ± 0.2	637 ± 24	0.90 ± 0.1	−11.9 ± 0.9
NEAT1–NEAT3 + VHH6	35.0 ± 0.1	0.33 ± 0.05	0.094 ± 0.002	ND	ND
Linker-NEAT3 + VHH35	6.6 ± 0.1	0.35 ± 0.01	0.53 ± 0.02	0.98 ± 0.01	−16.0 ± 0.3
NEAT3 + VHH35	3.9 ± 0.1	91.8 ± 1.1	237.2 ± 0.3	1.02 ± 0.1	−17.0 ± 0.5
Linker-NEAT3 + heme^b^	ND	ND	ND	1.18 ± 0.01	−14.5 ± 1.1

Abbreviation: ND, not determined.

a Mean of two independent measurements ± standard deviation.

b The experiment was performed without VHH for reference of the heme binding to linker-NEAT3.

In a separate experiment, ITC was performed by filling the calorimeter’s cell with the antigen while the antibody was loaded in the syringe as described in the Experimental procedures section (Table 1; Figs. 2*B* and S1*B*). In this experiment, it was also clear that VHH6 and VHH35 displayed high affinity for IsdH linker-NEAT3. The affinity was so high for this technique that precluded the accurate determination of *K*_*D*_ in this experiment. On the contrary, the values of *ΔH* were determined with a high degree of confidence because the first few injections produced constant values of exothermic heat. The values obtained for VHH6 and VHH35 were −17.1 ± 0.2 and −16.04 ± 0.31 kcal mol^−1^, respectively, indicating that the binding was enthalpy driven in both antibodies. When the antigen employed was NEAT3, the affinity dropped significantly, as seen from the change in the slope of the binding isotherm (Table 1; Figs. 2*D* and S1*D*), and consistent with the aforementioned SPR experiments.

Collectively, SPR and ITC data revealed that VHH6 and VHH35 recognized IsdH with similar properties. We note the importance of the linker region for the high affinity in both antibodies. Since VHHs tend to bind to hydrophobic clefts and pockets, such as the heme-binding pocket in NEAT3, we hypothesized that these VHHs could block heme binding, and we proceed to verify it.

### Blocking the binding of heme

**Figure 3:**
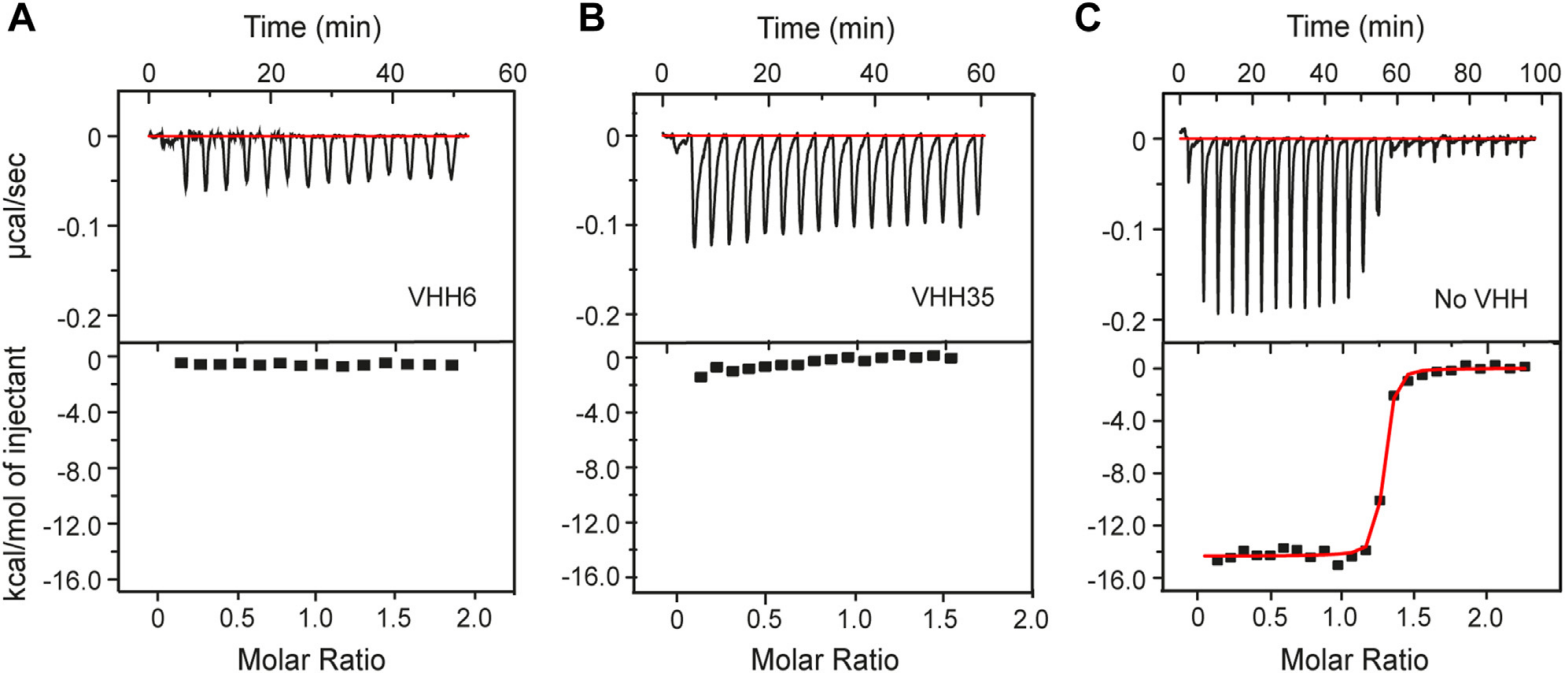
**VHHs inhibit the capture of heme by IsdH protein.** Titration of heme to IsdH linker-NEAT3 in the presence of (*A*) VHH6, (*B*) VHH35, or (*C*) in the absence of VHH. The titrations were performed in a VP-ITC instrument as described in the Experimental procedures section. The thermodynamic parameters of the experiment in the absence of VHH parameters are given in Table 1. ITC, isothermal titration calorimetry; NEAT, NEAr-iron transporter; VHH, variable domain of heavy chain of heavy chain–only antibody.

**Figure 4:**
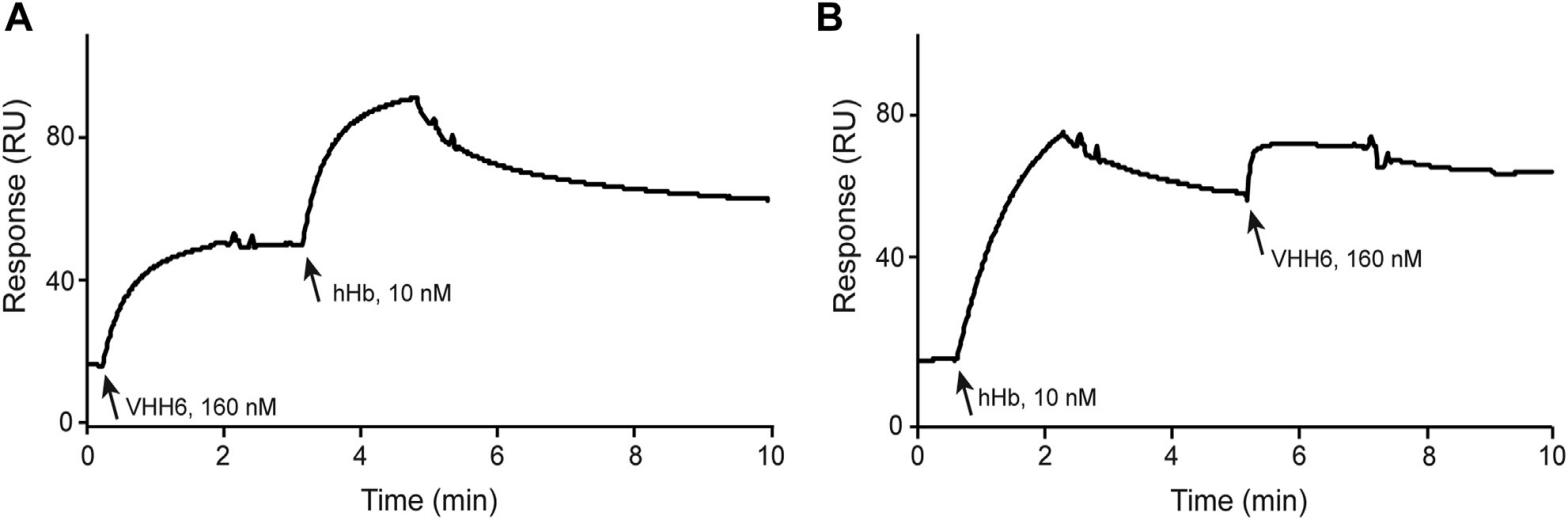
**Competitive binding of VHH6 and hemoglobin to IsdH.** The addition of analytes (VHH6 or hHb) is indicated with *arrows*, and their concentration is also shown. *A*, VHH6 was flowed at 160 nM and, 3 min later, hHb was flowed at 10 nM. This experiment thus corresponds to the binding of hHb to the complex between IsdH NEAT1–NEAT3 and VHH6. *B*, hHb was first injected at 10 nM and, 5 min later, VHH6 was injected at 160 nM. These experiments were performed in the manual run mode. hHb, human hemoglobin; VHH, variable domain of heavy chain of heavy chain–only antibody.

To investigate whether the VHH obtained could inhibit the acquisition of heme binding by the IsdH linker-NEAT3, a competitive binding experiment was performed by ITC (using SPR was highly problematic because of the tendency of heme to bind nonspecifically to the chip). In the ITC experiment, heme was loaded in the syringe and injected stepwise into the cell filled with linker-NEAT3 alone or with linker-NEAT3 bound to VHH6 or VHH35 (Fig. 3). By comparing the binding of heme to linker-NEAT3 with the binding of heme to linker-NEAT3 in complex with VHH, it would be possible to determine if the antibodies have the ability to block heme from binding to IsdH. The binding of heme to the unbound linker-NEAT3 is enthalpy driven with a ΔH of −14.5 ± 1.1 kcal mol^−1^ (Table 1 and Fig. 3*C*). In contrast, the injection of heme to IsdH linker-NEAT3 in complex with VHH6 or VHH35 did not generate significant exothermic heat after subtracting the dilution heat (Fig. 3, *A* and *B*). When comparing heme binding to IsdH linker-NEAT3 with heme binding to the complex containing VHH, it was concluded that heme could not bind to IsdH in the presence of the antibodies. Importantly, VHH6 and VHH35 could not bind to IsdH linker-NEAT3 in the heme-bound form (Fig. S2). These set of experiments clearly indicated that VHH6 and VHH35 blocked the binding of heme to NEAT3. Since both VHH6 and VHH35 shared 98% identity and almost identical binding properties, heretofore only VHH6 was employed for additional characterization. We also noticed that VHH6 could not block the binding of hHb and vice versa (*i.e.*, Hb could not block the binding of VHH6), as determined in the SPR competitive assays of Figure 4. In order to rationalize the observations made so far, we sought to obtain high-resolution structural data.

### Structure of VHH6 in complex with IsdH linker-NEAT3

**Figure 5:**
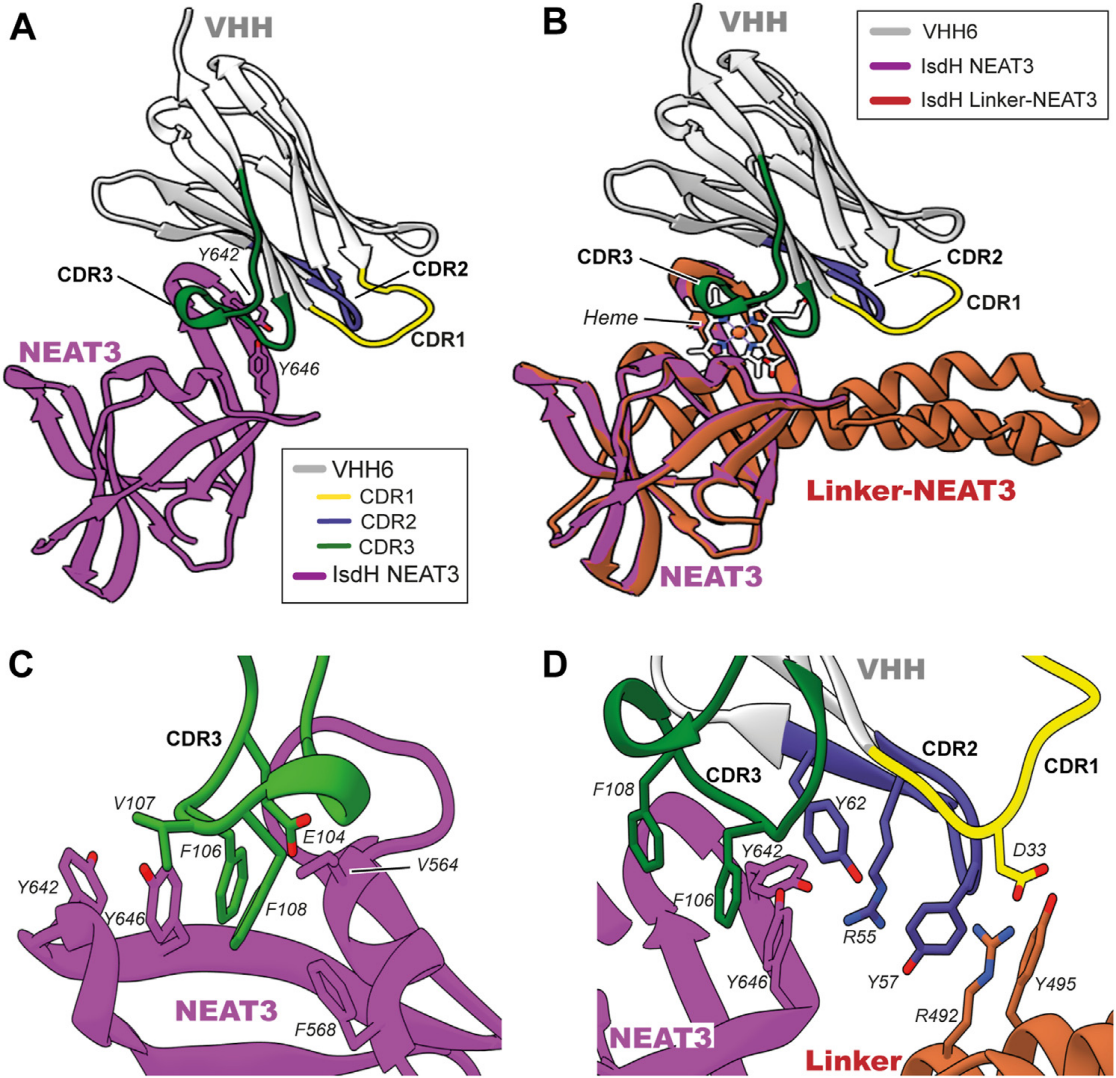
**Crystal structure of the complex of VHH6 with IsdH-NEAT3 at 1.65 Å resolution.***A*, VHH6 bound to IsdH NEAT3. VHH6 is shown in *white* with the CDR1, CDR2, and CDR3 depicted in *yellow*, *blue*, and *dark green* colors, respectively. *B*, structure of IsdH NEAT3-VHH6 superimposed to the structure of IsdH linker-NEAT3 bound to heme (RMSD = 0.866 Å) (Protein Data Bank entry code: 7XLD). IsdH NEAT3 is shown in *magenta*, and the superposed IsdH linker-NEAT3 bound to heme is depicted in *orange*. The heme appears in *white*. Relevant residues are indicated. *C*, close-up view of the proposed recognition area in the vicinity of the CDR3. *D*, detail of the proposed recognition site of CDR1–2. The figure was prepared with Chimera (53). CDR, complementary-determining region; NEAT, NEAr-iron transporter; VHH, variable domain of heavy chain of heavy chain–only antibody.

The structure of VHH6 in complex with IsdH linker-NEAT3 was obtained by X-ray crystallography (Fig. 5). The structure was determined at 1.65 Å, revealing that the antibody adopted a standard immunoglobulin-like with the corresponding three CDR loops protruding from it, and in particular, with the CDR3 interacting directly with the heme-binding pocket of the NEAT3 domain of IsdH (Fig. 5, *A* and *B*). In this loop, phenylalanine residues 106 and 108 of the VHH established interactions with Y646 of IsdH NEAT3. This tyrosine residue of NEAT3 is essential to coordinate the iron atom in heme (24, 25). There were also possible interactions of the CDR3 through residues V105 and E104 of the heme-binding pocket (Fig. 5*C*). The position and interactions of CDR3 with NEAT3 explain why VHH6 blocked the binding of heme to IsdH, since both CDR3 and heme competed for the same region of the receptor. Residues Y62 and R55 of the CDR2 of the antibody engaged in interactions with the backbone of the heme-binding pocket. CDR1 did not seem to interact with NEAT3.

We note that the linker of IsdH was not observed in the structure determined, and the reason was that the linker domain was excised during the weeks necessary to obtain quality crystals amenable to X-ray analysis. The same observation was made in a previous study in which the structure of linker-NEAT3 (heme-unbound form) was also pursued (26). Because the linker domain was not observed, we superimposed the NEAT3 domain of the new structure with the NEAT3 domain in the structure of IsdH linker-NEAT3 with heme bound to gain structural insight (26) (Fig. 5, *B* and *D*). From that comparison, we could estimate possible interactions of VHH6 with the linker region. Both structures containing NEAT3 shared an RMSD of 0.9 Å, and therefore, we can conclude that despite the very different nature of the crystals, NEAT3 has conserved a very similar structure. In this model, no residue from CDR3 appears to interact with the linker domain, whereas residue Y57 of CDR2 was located within interaction distance of residue Y465 in the linker. Residue D33 of CDR1 was suggested to interact with residue R492 of the linker (Fig. 5*D*).

### MD simulations of VHH6 in complex with IsdH linker-NEAT3

**Figure 6:**
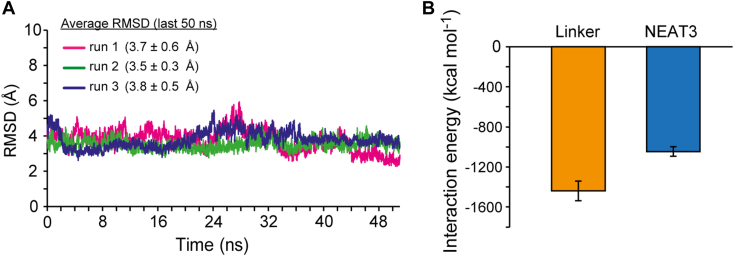
**Molecular dynamics (MD) simulations and interaction energy between IsdH linker-NEAT3 and VHH6.***A*, Cα-RMSD (in Å) calculated from the last 50 ns in three different trajectories. *B*, interaction energy between IsdH linker-NEAT3 domain and VHH6. Interaction energy (kcal mol^−1^) was defined as the sum of van der Waals and Coulomb interactions as determined in GROMACS 2016.3 using the CHARMM36m force field. *Bars* correspond to the data obtained when employing residues of the linker or residues of NEAT3 (in *orange* and *blue*, respectively). Error bars correspond to standard deviation. NEAT, NEAr-iron transporter; VHH, variable domain of heavy chain of heavy chain–only antibody.

It has been shown by SPR and ITC that the affinity of this antibody was dramatically decreased when interacting with NEAT3 alone, so the linker domain is essential for the recognition. The analysis of the interaction of VHH with only the linker domain will be challenging. To corroborate our findings, MD simulations of the complex VHH6–IsdH linker-NEAT3 were performed to analyze the interaction energies of the IsdH NEAT3 and linker domains (Fig. 6). MD simulations were performed using the crystal structure of IsdH NEAT3 in complex with VHH6, obtained herein. The linker domain was docked in the VHH-IsdH NEAT3 complex using the previous published crystal structure of IsdH linker-NEAT3 bound to heme (26). The RMSD of the last 50 ns was stable, without prominent fluctuations in the three independent runs with a value of 3.7 ± 0.6, 3.5 ± 0.3, and 3.8 ± 0.5 Å, respectively, and could be used for analysis (Figs. 6*A* and S3). The interaction energy of the last 50 ns of the three runs of simulations was analyzed for the interaction of linker or NEAT3 with the antibody VHH6 (Fig. 6*B*).

The MD simulations revealed that the contribution of each domain, linker, and NEAT3 to binding was relevant (Fig. 6*B*). SPR and ITC data demonstrated that the absence of the linker had a dramatic impact in the recognition by the VHH, which was in agreement with the results of the simulations. VHH6 recognizes linker and NEAT3, the energetic contributions of the interaction with each domains being similar to each other, and where the disappearance of one of the domains would decrease the affinity.

### Analysis of the epitope recognition mechanism by mutagenesis (alanine scanning)

**Figure 7:**
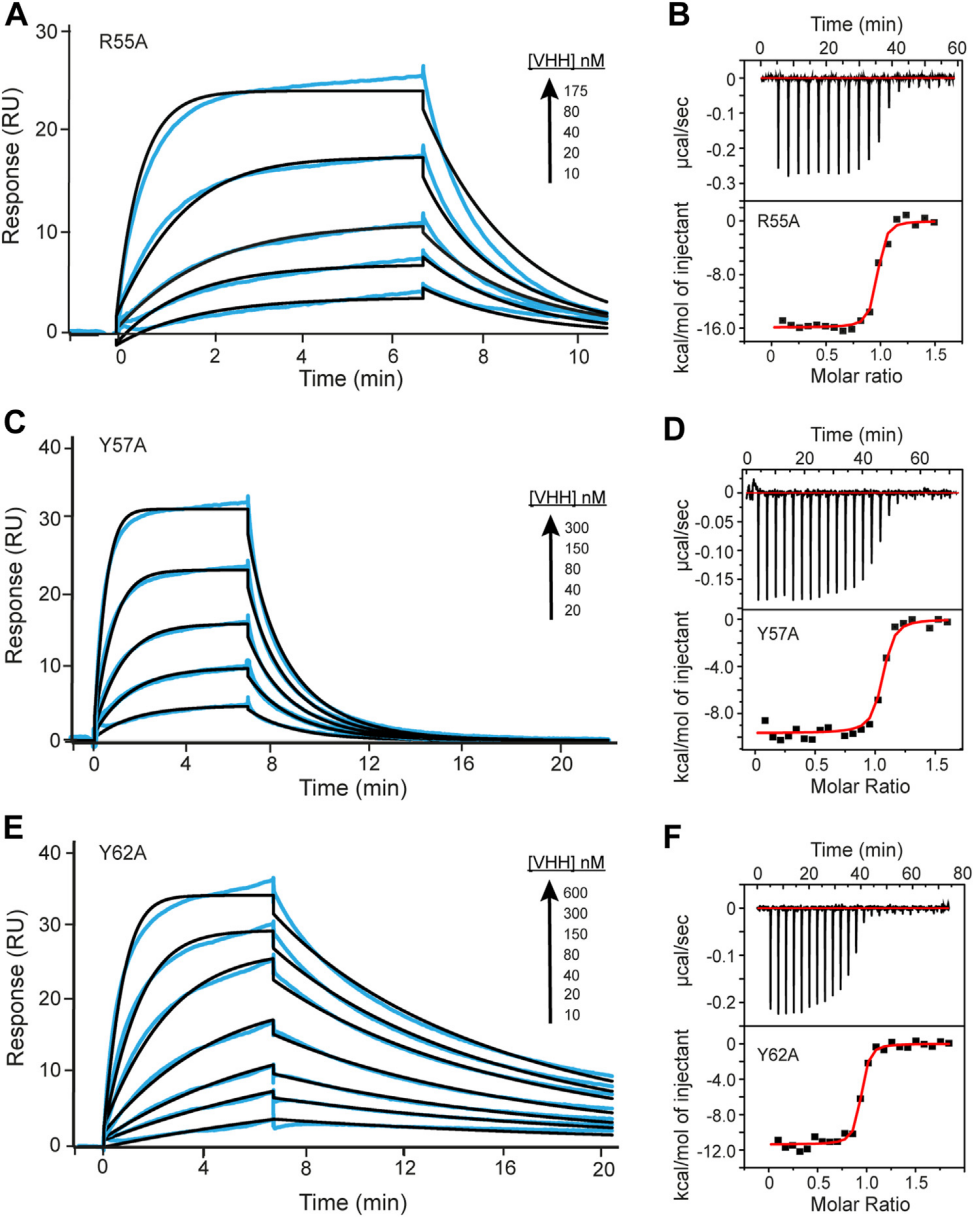
**Binding of alanine mutants of VHH6 to IsdH linker-NEAT3.***Left* and *right panels* correspond to data obtained by SPR and ITC, respectively. The following mutants of VHH were examined: (*A* and *B*) R55A; (*C* and *D*) Y57A; and (*E* and *F*) Y62A. Experiments employing ITC and SPR were performed and analyzed as described in the Experimental procedures section. The thermodynamic and kinetic parameters determined in this experiment are given in Table 2. ITC, isothermal titration calorimetry; NEAT, NEAr-iron transporter; SPR, surface plasmon resonance; VHH, variable domain of heavy chain of heavy chain–only antibody.

An extensive analysis of the epitope recognition was performed by alanine scanning. From the crystal structure, potential residues of VHH6 essential for the interaction with IsdH were changed to Ala, and their binding properties were analyzed by SPR and ITC using IsdH linker-NEAT3 (Table 2). The mutation of the residue Y57 from the CDR2 accelerates the dissociation and leads to a loss of affinity by ∼200-fold (Table 2 and Fig. 7). Interestingly, the *k*_off_ for VHH6 Y57A binding to linker-NEAT3 was 144 s^−1^, and the *K*_*D*_ value was 114 nM, whereas for the binding of WT VHH6 to NEAT3 without linker domain, the dissociation was 235 × 10^−4^ s^−1^ and the affinity 638 nM (Tables 1 and 2). The effect of the Y57 mutation led to drastic changes in the dissociation constant and in the affinity to the effect of the lack of the linker domain. Y57 is a key residue to recognize the linker region, and the interaction of Y57 with the linker residue Y495 seemed the most important for the affinity of VHH6. Mutation of the residues R55 and Y62 from CDR2 also were conducive to a decreased affinity of approximately 67- and 86-fold, respectively. These residues were important for the recognition of the antigen through interactions with the NEAT3 domain as seen in the crystal structure (Table 2; Figs. 5*D* and 7). The mutation of the other selected residues in CDR3 and CDR1 (Table 2 and Fig. S4) lead to a decrease in the binding affinity but compared with the mutation of Y57, R55, and Y62, the change was not as relevant. In the majority of VHH interactions, the CDR with the higher affinity interactions is usually the third loop (20); however, in the interaction of VHH6, it seems that CDR2 was contributing the most to the affinity and to epitope recognition (Table 2). MD simulations (Fig. S5) showed that residues such as Y57 and D33 of the antibody remained in proximity of residues of the linker region during the simulations, further strengthening the models constructed in Figure 5, *B* and *D*.

**Table 2:** Binding parameters of WT VHH6 and alanine mutants to IsdH linker-NEAT3^a^

Protein	SPR				ITC		
	*k*_on_ (×10^4^ M^−1^s^−1^)	*k*_off_ (×10^−4^ s^−1^)	*K*_*D*_ (nM)	Relative *K*_D_^b^	n (mol:mol)	*ΔH* (kcal mol^−1^)	*ΔΔH*^b^ (kcal mol^−1^)
WT	8.1 ± 0.1	0.42 ± 0.02	0.53 ± 0.02	1	1.1 ± 0.03	−17.1 ± 0.2	0
D33A	6.2 ± 0.1	1.3 ± 0.01	2.08 ± 0.01	3.9	1.01 ± 0.02	−14.7 ± 0.1	2.4
R55A	28.4 ± 0.1	85.5 ± 5.1	25.44 ± 3.0	48	0.94 ± 0.01	−15.9 ± 0.1	1.2
Y57A	12.6 ± 0.3	143.9 ± 1.2	114.1 ± 3.3	215	1.03 ± 0.01	−9.7 ± 0.1	7.5
Y62A	4.6 ± 0.1	20.7 ± 0.1	45.4 ± 0.3	86	0.93 ± 0.02	−11.1 ± 0.3	6
E104A	15.1 ± 0.3	14.2 ± 0.2	6.4 ± 3.0	12	0.99 ± 0.01	−13.6 ± 0.5	3.5
F106A	9.9 ± 0.1	3.1± 0.1	3.1 ± 0.2	5.8	1.01 ± 0.01	−16.6 ± 0.1	0.5
V107A	10.9 ± 0.1	10.5 ± 0.1	9.7 ± 0.1	18	0.96 ± 0.02	−15.9 ± 0.1	1.2
F108A	18.9 ± 2.5	4.9 ± 0.6	2.6 ± 0.1	4.9	0.97 ± 0.01	−19.3 ± 1.1	−2.2

a Mean of two independent measurements ± standard deviation.

b Relative *K*_*D*_ and Δ*ΔH* were calculated with respect to the values of WT VHH.

### VHH6 binding to IsdB linker-NEAT2

In the cell wall surface of *S. aureu*s, not only IsdH but also IsdB recognize Hb and extract its heme moiety (17, 27). Even if heme acquisition is completely blocked in one of the receptors, heme could still be extracted from Hb by the second receptor, and the bacterium would in principle have sufficient resources to satisfy its nutritional requirements of iron for growth. To generate an effective agent blocking Isd-mediated heme import of iron, it would be desirable to block heme acquisition simultaneously for both, IsdH and IsdB receptors. IsdH and IsdB share ∼65% of sequence identity and even greater identity in the heme-binding pocket region (75%) (28, 29), and their structures are also similar (RMSD ∼0.8 Å). Therefore, we hypothesized that VHH6 could recognize not only IsdH linker-NEAT3 but also the equivalent region in IsdB. If that was indeed the case, this antibody could block heme receptor also in IsdB raising expectations for this strategy.

The binding of VHH6 to IsdB linker-NEAT2 was analyzed and compared with the binding to IsdH linker-NEAT3 by ITC and SPR (Table 3 and Fig. S6). VHH6 binds to IsdH linker-NEAT3 and IsdB linker-NEAT2 with nanomolar affinity. The enthalpy obtained by ITC and the dissociation and association constants obtained by SPR for the VHH6 binding to the two Isd proteins were similar, indicating that the antibody recognized both receptors.

**Table 3:** Binding parameters of VHH6 to IsdB linker-NEAT2^a^

Protein	SPR			ITC	
	*k*_on_ (×10^4^ M^−1^ s^−1^)	*k*_off_ (×10^−4^ s^−1^)	*K*_*D*_ (nM)	n (mol:mol)	*ΔH* (kcal mol^−1^)
IsdH linker-NEAT3	8.1 ± 0.04	0.42 ± 0.02	0.53 ± 0.02	1.1 ± 0.03	−17.1 ± 0.2
IsdB linker-NEAT2	16.0 ± 0.1	0.4 ± 0.05	0.25 ± 0.03	0.8 ± 0.01	−24.2 ± 0.8

a Mean of two independent measurements ± standard deviation.

**Figure 8:**
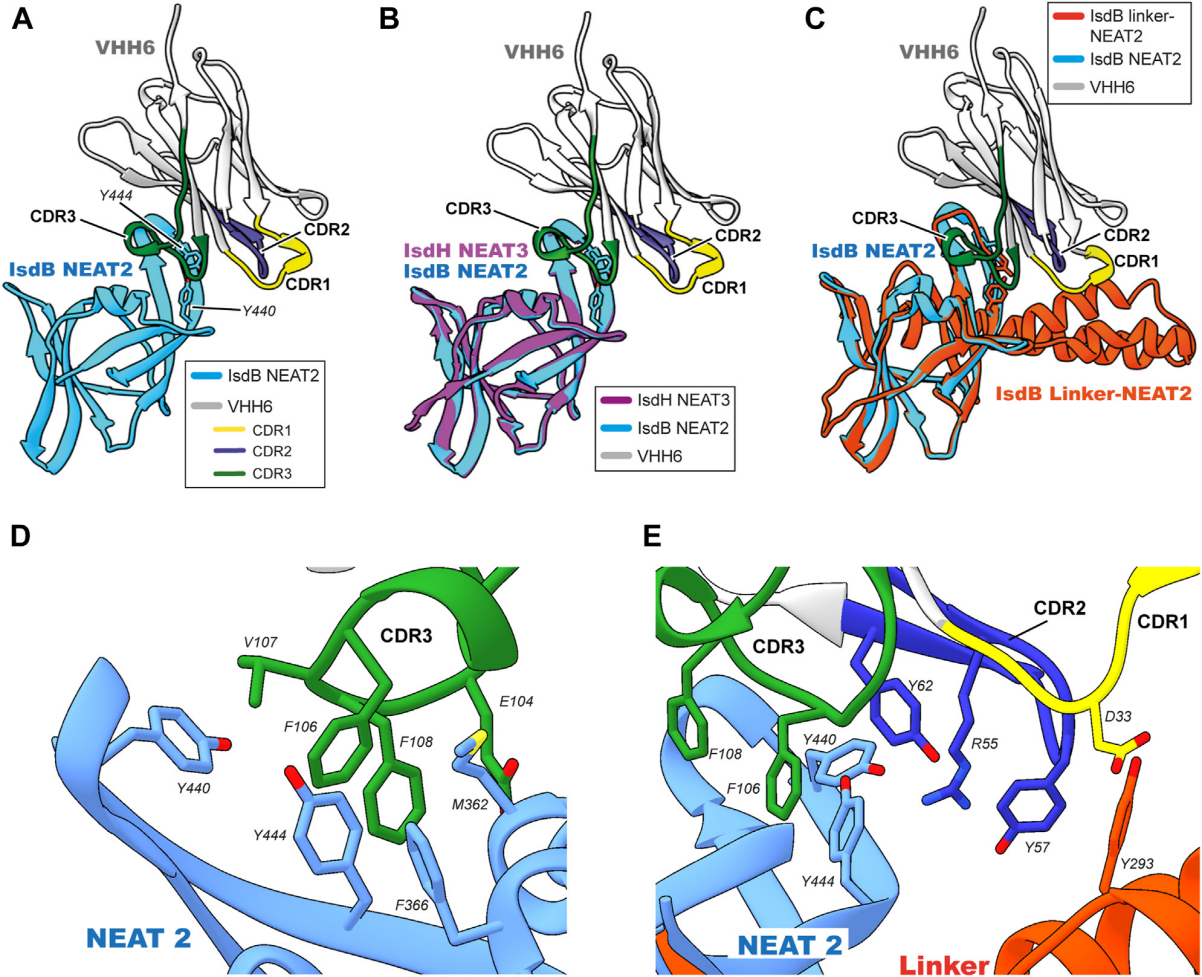
**Crystal structure of the complex of VHH6 with IsdB NEAT2 at 1.70 Å resolution.***A*, structure of VHH6 bound to IsdB NEAT2. VHH6 is shown in *white* with their CDR1, 2, and 3 depicted in *yellow*, *dark blue*, and *dark green*, respectively. IsdB NEAT2 is shown in *light blue*. *B*, IsdB NEAT2 in complex with VHH6 superposed to the structure of IsdH NEAT3 (RMSD = 0.812 Å). The superposed IsdH NEAT3 and IsdB linker-NEAT2 (Protein Data Bank entry code: 5VMM (27)) are shown in *magenta* and *orange*, respectively. *C*, IsdB NEAT2 in complex with VHH6 superposed with the structure of IsdB linker-NEAT2 (Protein Data Bank entry code: 5VMM (27), RMSD = 0.757 Å). Relevant residues are indicated. *D*, close-up view of the interaction region in the vicinity of the CDR3. *E*, detailed view of the proposed recognition site mediated by CDR1 and CDR2. This figure was prepared using UCSF Chimera (53). CDR, complementary-determining region; NEAT, NEAr-iron transporter; VHH, variable domain of heavy chain of heavy chain–only antibody.

The crystal structure of VHH6 bound to IsdB linker-NEAT2 was obtained by X-ray crystallography (Fig. 8). The structure was determined at a resolution of 1.70 Å. Similarly to IsdH linker-NEAT3, the linker of IsdB was cleaved off during the weeks leading to crystal formation. The structure of IsdH NEAT3 (Protein Data Bank [PDB] entry code: 7W81 (26), Fig. 8*B*) and the structure of IsdB linker-NEAT2 (PDB entry code: 5VMM (27), Fig. 8*C*) were superposed with the coordinates of the structure of IsdB NEAT2 in complex with VHH6 (this work; PDB entry code: 7XLI) to compare and analyze the interactions. The structure of VHH6 bound to IsdB and IsdH was very similar with an RMSD of 0.812 Å. The CDR3 is located inside the IsdB NEAT2 heme-binding pocket, and the CDR2 seemed to maintain similar interactions with the linker of IsdB (Fig. 8, *B* and *C*).

**Figure 9:**
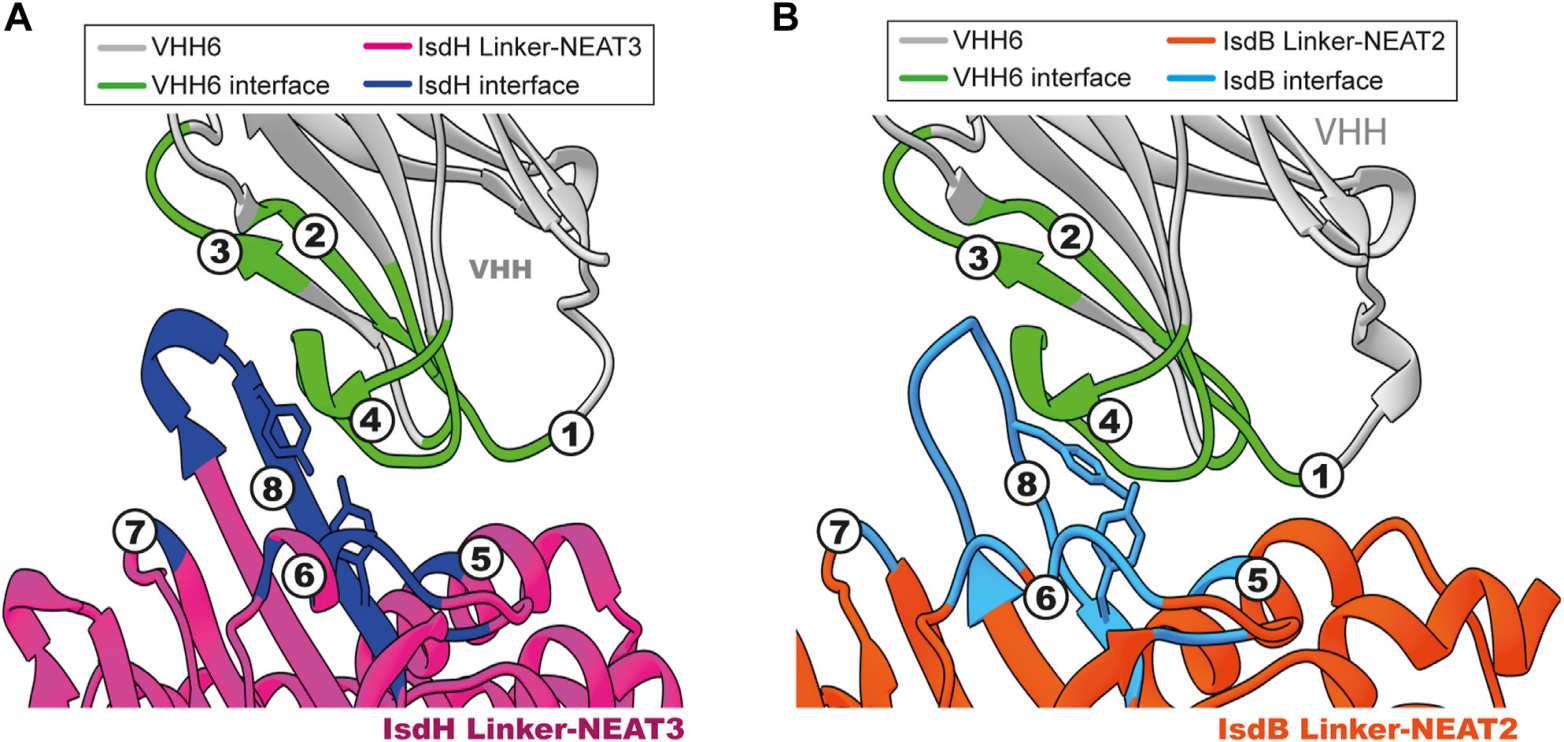
**Interaction regions of VHH6 bound to the heme-binding domain of IsdH or IsdB.***A*, close-up view of the binding interface between VHH6 and IsdH. The *circled numbers* indicated the interface segments belonging to VHH6 (1: 33-DNFII-37; 2: 50-GVSFLRKY-57; 3: 62-YYAES-66; 4: 102-DREGFVFEQGMD-113; and 5: Y495) or IsdH (6: 557-SEENSESVMDGF-568; 7: Y593; and 8: 637-VANIGYEGQYHV-648). The antibody is depicted in *white* except the binding interface, which is shown in *green*. IsdH linker-NEAT3 is shown in *magenta* with the interface in *dark blue*. *B*, detailed view of the binding interface between IsdB and VHH6. The *circled numbers* indicating interfacial regions of VHH6 (1: 33-DNFI-36; 2: 50-GVSFLRKY-57; 3: 62-YYAES-66; and 4: 102-DREGFVFEQGMD-113) and IsdB (5: Y243; 6: 354-ESVENNESMMDTF-366; 7: Y391; and 8: 433-VHVKTICYDGQYHV-446). The color scheme of the antibody is the same as in panel (*A*). IsdB linkerNEAT2 is shown in *orange* with the interface in *light blue*. The figure was prepared using UCSF Chimera (53). VHH, variable domain of heavy chain of heavy chain–only antibody.

The comparison of the interaction interfaces of VHH6–IsdH and VHH6–IsdB would contribute to the understanding of the bispecific binding. An analysis of the complex crystal structures of VHH6 with IsdH and VHH6 with IsdB using the protein interfaces, surfaces, and assemblies service at the European Bioinformatics Institute (30) revealed that the interface of the VHH6 interacting with IsdB and IsdH was nearly identical (Fig. 9). By representing and comparing the residues located at the interaction interface of VHH6 with IsdH, and that of VHH6 with IsdB in their corresponding complex crystal structures (Fig. 9, *A* and *B*), it was possible to determine that the interfaces of IsdB and IsdH recognized by VHH6 are very similar (67% identity). In addition, the most relevant residue for VHH recognition revealed by alanine scanning (Y57 from CDR2) would interact with Y495 from IsdH and with Y243 from IsdB, both residues located structurally in the same position in the linker domains of IsdH and IsdB in complex with VHH. It is clear that most interactions of the VHH6 would be maintained in the recognition of both proteins, including the essential contribution of Y57.

### Inhibition of growth assay

**Figure 10:**
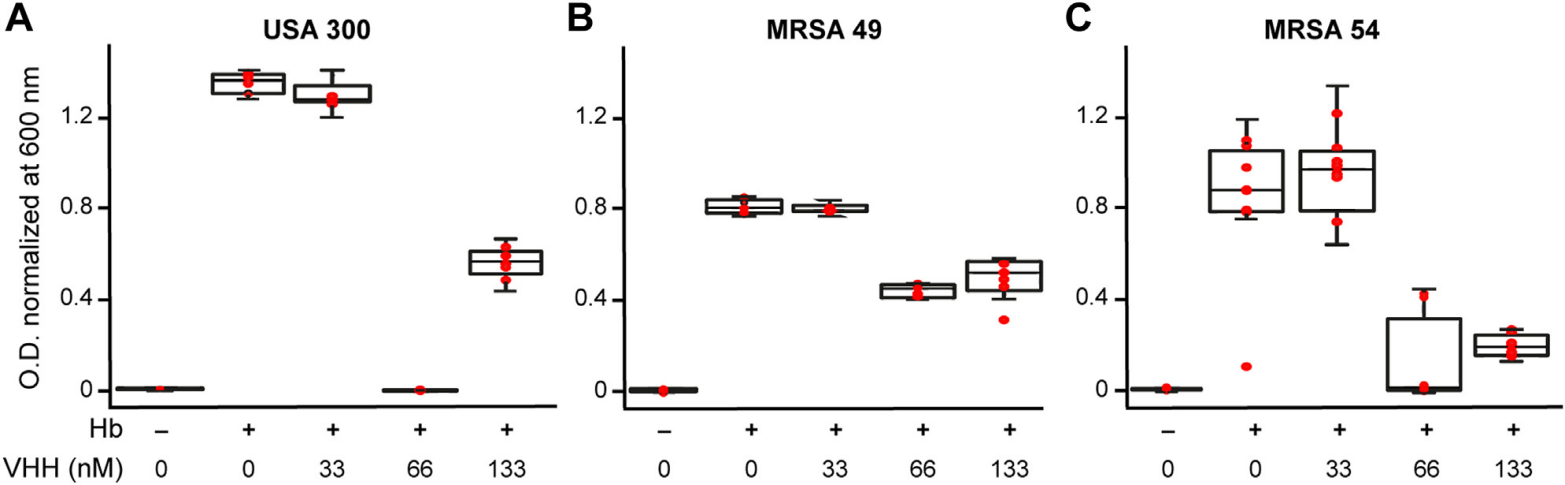
***Staphylococcus aureus* growth inhibition assay.** Different MRSA strains were cultured in the presence of 2 μM EDDHA (a strong metal chelator), 20 nM hHb as the unique iron source, and increasing concentrations of VHH6 as described in the Experimental procedures section. For each assay, the absorbance at 600 nm was measured and normalized to 1.0. The presence or the absence of hHb in the medium is indicated with a + symbol or − symbol, respectively. The panels correspond to the growth assay of (*A*) MRSA strain USA300 (CP000255) (51), (*B*) MRSA strain MRSA49 (KUH180062; AP020321 (51)), and (*C*) MRSA strain MRSA54 (KUH180129; AP020323 (51)). Individual measurements are indicated with *red dots*, and the median and standard deviation error are indicated with *black lines* in a box-and-whisker representation. EDDHA, ethylenediamine-*N*, *N*′-bis (2-hydroxyphenylacetic acid); hHb, human hemoglobin; MRSA, methicillin-resistant *Staphylococcus aureus.*

The high affinity of VHH6 to both IsdH and IsdB, their similar binding mechanism and similar structures, and the capability to block heme binding to IsdH are all pointing at the possibility that VHH6 could be a candidate to inhibit heme acquisition by the bacterium. To verify this hypothesis, we evaluated the effect of VHH6 in the growth of *S. aureus* at the cellular level. Three different strains of *S. aureus* directly obtained from patients suffering from Staphylococcal infection where incubated, and bacterial growth was measured by absorbance as described in the Experimental procedures section. The medium in which the experiments were carried out contained Hb as the unique source of iron, and the growth of these strains of *S. aureus* without Hb was also monitored as control. From the experiments, it is clear that increasing concentrations of VHH6 in the medium (nanomolar level) lead to a moderation and in some cases complete inhibition in the growth of *S. aureus* (Fig. 10). The inhibitory responses were not homogeneous in all strains, and for example, USA300 showed a peculiar dose–response profile in which part of the inhibitory effect was lost at the greatest concentration of VHH examined, whereas for strains MRSA49 and MRSA54, there was a saturation in the extent of inhibition observed. However, these data also indicated that, collectively, the presence of VHH6 had an inhibitory effect on the bacterial growth in all three strains examined. Despite individual peculiarities, we concluded that the presence of VHH6 exerted a clear inhibitory effect on the bacterial growth in all three strains examined, and that the cause was specifically the presence of the VHH in an iron-limited environment (Fig. S7).

## Discussion

Two VHH-recognizing IsdH linker-NEAT3s, number 6 and number 35, were selected for characterization. Because of the similar binding characteristics of VHH6 and VHH35, it can be concluded that their differences in the first two residues does not have an effect on the interaction with the antigen. The majority of camelid VHH stablish interactions with the antigen mainly though the CDR3 (20). However, in the VHH6 obtained in this research, the CDR2 has the most relevance in the antigen recognition. The SPR, ITC, and complex crystal structure of the VHH6 interacting with IsdH demonstrated that VHH6 recognizes the NEAT3 and the linker domain with CDR3 and CDR2, respectively. The CDR3 is located within the binding pocket of the IsdH NEAT3 and IsdB NEAT2 domain, and there would be no space for heme to bind to IsdH or IsdB, which would explain the mechanism of heme-binding inhibition. From the models in Figure 5 and MD simulations (Fig. S5), we hypothesized that Y57 CDR2 of VHH6 interacts with Y495 of IsdH linker, and this interaction would be crucial for the high-affinity complex. This Y495 residue of the IsdH linker has been proven to be involved in the interaction and destabilization of the F-helix of rHb (31, 32). In VHH6, Y57 is a key residue for the recognition of the linker, and it could be important in the inhibition of the interaction with Hb and in the inhibition of heme extraction. Therefore, the effectivity of the inhibition of the heme binding by the VHH6 arises from the recognition of both linker and NEAT domains.

It was demonstrated that the VHH6 recognizes IsdH and IsdB. The sequence and structural similarities of both proteins (28, 29) would ensure that the residues involved in the contact with VHH6 are preserved, which would explain the mechanism by which VHH can interact with both proteins maintaining the nanomolar affinity. The recognition of different Isd proteins by antibodies was previously reported by other researchers. In previous investigations, human monoclonal antibodies extracted from infected patients that could recognize IsdH, IsdB, and IsdA were analyzed (33, 34). These antibodies interact mainly with the heme-binding pocket of the NEAT domains but do not bind to the linker domains. The recognition of different proteins from the Isd system by antibodies is possible because of the interactions with the conserved sequences and structures of the heme-binding pockets of the NEAT domains but the recognition of the linker domain stablished by VHH6 might be relevant for the inhibition of heme acquisition from Hb and for preventing bacterial growth. The inhibition of growth of pathogenic strains of MRSA *S. aureus* by the VHH6 antibody obtained herein could be an indicator that this antibody has the potential to be used, after humanization, for therapy. In summary, the first antibody recognizing IsdH and IsdB with proven growth inhibition capability is reported in this study.

## Experimental procedures

### Cloning of Isd proteins

The gene corresponding to full-length IsdH was amplified from genomic DNA of *S. aureus* Mu50 (17) and introduced into the expression vector pET28b containing an N-terminal His_6_-SUMO tag using the HiFi assembly procedure (New England BioLabs). To prepare the linker-NEAT3 construct, BamH1 and XhoI restriction sites were introduced in the region comprising Asn476 to Gln664 of the vector containing full-length IsdH, using a KOD-Plus mutagenesis kit (Toyobo) according to the manufacturer's instructions. The restriction fragment, containing linker-NEAT3, was introduced in a pET28-His_6_-SUMO vector. For NEAT3, a pET28b expression vector containing an N-terminal His_6_ tag followed by a thrombin cleavage site and the corresponding NEAT3 region from Gly534 to Gln664 were obtained as described previously (35). The gene of IsdB linker-NEAT2 (from residue Lys247 to Ala453) was optimized for expression in *Escherichia coli* and introduced into a pET28-His_6_-SUMO vector using HiFi assembly kit (New England Biolabs).

### Expression of Isd proteins in the heme-bound and heme-unbound forms

*E. coli* C43 (DE3) competent cells were independently transformed with the corresponding pET28 vectors, containing IsdH NEAT1-NEAT3, IsdH linker-NEAT3, IsdH NEAT3, or IsdB linker-NEAT2. Transformed cells were screened on Petri dishes with LB–agar containing kanamycin (50 μg ml^−1^; Wako). Expression of the heme-free proteins was carried out in M9 minimal medium supplemented with kanamycin (5 mg l^−1^) as described previously (16). To produce the heme-bound forms, transformed cells were grown in LB medium supplemented with 100 μM FeCl3 (Wako) and incubated at 37 °C (26). When the absorbance at 600 nm reached a value of 0.4, protein expression was induced by addition of 0.5 mM IPTG and cells were incubated for 20 h at 28 °C.

### Purification of Isd proteins

After protein expression, the cells were harvested by centrifugation at 4 °C for 10 min at 8000*g* (Tomy) and then resuspended in PBS (10.13 mM Na_2_HPO_4_·12 H_2_O, 1.76 mM KH_2_PO_4_, 2.7 mM KCl, 137 mM NaCl at pH 7.4) containing 5 mM imidazole (Wako). The cell suspension was lysed with an ultrasonic sonicator on ice for 10 min with 0.5 s pulse (Tomy). The soluble fraction was separated by centrifugation for 30 min at 20,000 rpm (Tomy) at 4 °C, filtrated with a 0.22 μm sterile filter (Millex GP), and loaded into a gravity open column, equilibrated with PBS, containing 5 mM imidazole to perform immobilized-metal affinity chromatography (IMAC) using nickel–nitrilotriacetic acid (Ni–NTA) agarose (QIAGEN). The column was washed with 10 column volumes of PBS containing 15 mM imidazole. Proteins were eluted with 15 to 500 mM imidazole step gradient in PBS buffer. The fractions containing Isd proteins were collected.

To remove SUMO tag from IsdH NEAT1–NEAT3, IsdH linker-NEAT3, and IsdB linker-NEAT2, protein samples were separately incubated with Ulp1 protease for 20 h at 4 °C during dialysis in PBS. In the case of IsdH NEAT3 purification, the protein was incubated for 20 h at 4 °C with thrombin (Sigma–Aldrich) (1 U of thrombin per mg of protein) during dialysis in PBS. The cleaved His_6_-SUMO tag, UlpI protease, and His_6_-tag were separated from the target proteins by an additional gravity open column (IMAC) using Ni–NTA agarose. The column was equilibrated with PBS containing 15 mM imidazole, and IsdH NEAT1–NEAT3, IsdH linker-NEAT3, IsdH NEAT3, or IsdB linker-NEAT2 were independently collected in the flowthrough during washing with PBS containing 15 mM imidazole. Size-exclusion chromatography (SEC) using HiLoad 16/600 superdex 200-pg column (GE Healthcare) in an AKTA system equilibrated with PBS buffer was the last step of purification for all aforementioned proteins. The fractions containing the target proteins were collected. Purified protein was flash-frozen with liquid nitrogen and stored at −70 °C.

Concentration of IsdH NEAT1–NEAT3, IsdH linker-NEAT3, IsdH NEAT3, and IsdB linker-NEAT2 was measured on NanoDrop One (ThermoFisher Scientific) using molar extinction coefficients 89,620 cm^−1^ M^−1^, 21,890 cm^−1^ M^−1^, 15,930 cm^−1^ M^−1^, and 21,890 cm^−1^ M^−1^, respectively. Extinction coefficients were calculated based on their sequences using ProtParam Tool (ExPASy; Research Resource Identifier: SCR_018087). Protein purity after SEC was judged by SDS-PAGE and after staining with Coomassie brilliant blue R-250 (Wako) using Blue Protein Standard broad range (New England BioLabs) as a reference molecular weight marker.

### Expression and purification of Ulp1

The gene encoding UlpI with an N-terminal His_6_ tag in a pET28M vector was expressed in *E. coli* BL21(DE3). Expression was induced by addition of IPTG at 1 mM; after induction, the cells were grown for 20 h at 28 °C. The cells were harvested by centrifugation at 4 °C for 10 min at 8000*g*. Harvested cells were resuspended in 20 mM Tris buffer, pH 7.4, containing 300 mM NaCl and 20 mM imidazole and disrupted by sonication on ice for 10 min with 0.5 s pulse. The soluble portion was obtained by centrifugation (20,000 rpm for 30 min at 4 °C) and applied to a gravity open column to perform IMAC using Ni–NTA agarose. After washing the column with 10 column volumes of the same buffer, Ulp1 was eluted with an imidazole gradient (20–500 mM). The fractions containing Ulp1 were collected and subsequently loaded to Superdex 16/600 pg-75 column in an AKTA system equilibrated with 20 mM Tris buffer, pH 7.4, containing 300 mM NaCl. Protein purity was judged by SDS-PAGE using Blue Protein Standard broad range as a reference molecular weight marker. The protein was flash-frozen with liquid nitrogen and stored at −70 °C.

### Purification of hHb

Human methemoglobin as lyophilized powder was prepared in PBS at 2 mg/ml concentration and filtered with a 0.22 μm sterile filter (Millex GP). hHb was purified in an SEC using HiLoad 16/600 superdex 200-pg column in an AKTA system equilibrated with PBS buffer. The fractions containing hHb were collected and saturated with CO on ice for 10 min in order to obtain methemoglobin. Methemoglobin was always used fresh directly from purification.

The purity of hHb was corroborated by SDS-PAGE after SEC, using Blue Protein Standard broad range as a reference molecular weight marker. The concentration of hHb was determined by bicinchoninic acid protein assay using bovine serum albumin as standard (BCA protein assay kit; ThermoFisher Scientific). The absorbance at 562 nm was measured in a plate-reader EnSpire instrument (PerkinElmer).

### Antibody library construction and selection

An alpaca was immunized with IsdH linker-NEAT3 in the heme-depleted form. The immunization period was 5 weeks with a unique dose of a mix of different target antigens, included IsdH linker-NEAT3, per week. After confirmation of elevated antigenic reaction by ELISA, total RNA was extracted from lymphocytes using Trizol (ThermoFisher Scientific) according to the manufacturer’s protocol followed by complementary DNA synthesis using superscript III (ThermoFisher Scientific). VHH sequences were amplified from the complementary DNA by PCR using KOD-One and incorporated into a phagemid vector, resulting in the VHH-phage display library (21, 36).

The obtained library was electroporated into TG-1 *E. coli* cells, and phage production was induced with infection by M13KO7 helper phage. After overnight incubation, phage was precipitated from the supernatant with PEG/NaCl and resuspended in PBS. The antigen IsdH linker-NEAT3 was immobilized at 400 nM on immuno tube (ThermoFisher Scientific) by incubation at 4 °C overnight. After three times washing with PBS-T (0.05% Tween), the immuno tube was blocked by 3% skim milk in PBS. Subsequently, VHH-phage was added into the immuno tube and incubated at room temperature for 1 h. After ten times washing with PBS-T, the phage was eluted by incubation with 0.1 M glycine–HCl at pH 2.2 for 8 min and neutralized with 1 M Tris (pH 9.1) and used to infect HST02 *E. coli* cells. The infected *E. coli* cells were incubated, and grown cells were infected by helper phage M13KO7, and VHH-displaying phage was amplified overnight. Phage was precipitated from the supernatant with PEG/NaCl and used for subsequent round.

### VHH screening

After two rounds of selection for binding to IsdH linker-NEAT3, hit clones were surveyed by single-clone ELISA. *E. coli* cells infected by the eluted phages were picked up as single colonies and grown in 1 ml of 2× TY broth. Clones were infected by helper phage M13KO7 and incubated at 30 °C for overnight. The VHH-displaying phages were precipitated with PEG/NaCl and used for ELISA to select the clones with stronger binding to IsdH linker-NEAT3. The antigen was added at 400 nM into 96-well microtiter wells and incubated at 4 °C overnight. After three times of washing with PBS-T, the wells were blocked with 5% skim milk in PBS-T. Subsequently, the phage-displayed antibodies were added and incubated at room temperature for 1 h. Following three times washing with PBS-T, anti-M13 phage rabbit antibody conjugated with horseradish peroxidase and diluted with PBS-T containing 3% skim milk was added and incubated at room temperature for 1 h. After three times washing with PBS-T, tetramethyl-benzidine was added and incubated for 30 min, and the reaction was stopped with tetramethyl-benzidine stop buffer (COSMO-BIO). The sequences of hit clones that showed higher absorbance signal at 450 nm could recognize IsdH linker-NEAT3 and, therefore, were analyzed and chosen for further investigation. The determination of the framework regions and the CDRs was performed using the Kabat numbering and CDR scheme in the AbYsis server (37, 38).

### Antibody cloning, expression, and purification

The selected VHH genes, corresponding with hit clones 6 and 35, were amplified by PCR (ThermoFisher Scientific) using KOD-One polymerase and subcloned by HiFi assembly (New England Biolabs) into a pRA2 expression vector containing a PelB signal peptide, following the instructions from the manufacturer. Cells of *E. coli* BL21(DE3) were separately transformed with the resultant expression vectors and screened on Petri dishes with LB–agar containing ampicillin (50 μg ml^−1^; Wako) and grown in LB medium at 28 °C. Protein expression was induced by addition of 0.5 mM IPTG when the absorbance at 600 nm reached a value of 0.8, and cells were incubated for 20 h at 20 °C. The cells were harvested by centrifugation at 8000*g* for 10 min at 4 °C, and the cell pellet was resuspended in 20 mM Tris–HCl, 500 mM NaCl at pH 8.0 (Tris buffer), supplemented with 5 mM imidazole. The cell suspension was lysed with an ultrasonic homogenizer on ice for 10 min with 0.5 s pulse (Tomy). The soluble fraction was separated by centrifugation for 30 min at 20,000 rpm (Tomy) at 4 °C, filtrated with a 0.22 μm sterile filter (Millex GP), and loaded into a gravity open column to perform IMAC using Ni–NTA agarose. The column was washed with 10 column volumes of Tris buffer containing 5 mM imidazole. The antibody was eluted with a 100 to 500 mM imidazole step gradient in Tris buffer. The fractions containing VHH were collected and dialyzed in PBS for 20 h at 28 °C. The antibodies were further purified by SEC using HiLoad 16/600 superdex 75-pg column (GE Healthcare) equilibrated with PBS and in an AKTA system. The fractions containing the antibodies were collected to be used in further experiments. Concentrations of VHH were measured on NanoDrop One using molar extinction coefficient of 19,940 cm^−1^ M^−1^. Extinction coefficient was calculated based on the amino acid sequence using ProtParam Tool (ExPASy; Research Resource Identifier: SCR_018087). Protein purity after SEC was judged by SDS-PAGE after staining with Coomassie brilliant blue R-250 using Blue Protein Standard broad range as a reference molecular weight marker.

### Antibody single mutants for alanine scanning

Single mutants from VHH6 (D33A, R55A, Y57A, Y62A, E104A, V107A, F106A, and F108A) were obtained from the WT construct by single-mutation PCR with KOD-One polymerase in a thermocycler (ThermoFisher) and using the primers summarized in Table S2. Plasmids encoding the different mutants were individually transformed into *E. coli* BL21(DE3) and expressed and purified with the same protocol as the WT, described previously. The identity and purity of the VHH mutants were also determined as described previously.

### Antibody binding affinity

The antibody binding affinity was measured by SPR and ITC.

On SPR, IsdH NEAT1–NEAT3, IsdH linker-NEAT3, IsdH NEAT3, or IsdB linker-NEAT2 were independently immobilized in a CM5 chip (Cytiva) by amine coupling (39) with acetate at pH 4.5 (Cytiva) and with an immobilization level aim of 300 RU. The antibody binding was measured in a multicycle manner with concentrations of VHH ranging from 2.5 μM to 1.2 nM, and the regeneration steps were performed either with PBS-T (containing 0.005% Tween) or with 10 mM glycine–HCl at pH 2.0 (Cytiva). SPR was performed on a Biacore T200 (Cytiva), and the data were analyzed with BIAevaluation (Biacore AB) software. Experiments of SPR-binding affinity of the VHH6 single mutants to IsdH linker-NEAT3 were performed with the same protocol as for WT.

A SPR control experiment of VHH6 binding to heme-bound IsdH linker-NEAT3 on a Biacore 2000 (GE Healthcare) was performed by immobilizing linker-NEAT3 bound to heme by amine coupling in a CM5 chip with acetate at pH 4.5 with an immobilization level aim of 300 RU. The antibody binding was tested in a manual run at a concentration of VHH of 10 μM.

The ITC experiments were performed in an ITC200 (MicroCal). The antibodies (VHH6 or VHH35) and the antigens (IsdH linker-NEAT3, IsdH NEAT3, or IsdB linker-NEAT2) were dialyzed separately in PBS at 4 °C overnight. The cell was filled with antigen, and the syringe was loaded with VHH in a molar concentration ratio of 1:10 antigen:antibody. The concentrations used ranged from 100 to 6 μM for the antigen and from 1 mM to 60 μM for the VHH. The first injection of 0.5 μl (omitted from the analysis) was followed by 23 injections of 1.5 μl with 180 s intervals at a constant temperature of 25 °C. The titration syringe was continuously stirred at 750 rpm. The obtained data were fitted by nonlinear regression of the integrated data to a one-site binding model using ORIGIN (MicroCal). Experiments of ITC binding of the VHH6 single mutants to IsdH linker-NEAT3 were performed with the same protocol as for the WT. Control experiments of VHH binding to heme-bound IsdH linker-NEAT3 were performed by filling the cell with IsdH linker-NEAT3 bound to heme.

### Inhibition of heme binding by VHH

First, heme binding to IsdH linker-NEAT3 without VHH was measured on VP-ITC as a positive control (MicroCal). Proteins were dialyzed in PBS at 4 °C overnight. The cell was filled with 4 μM of heme-depleted IsdH linker-NEAT3, in PBS buffer containing 5% dimethyl sulfoxide, and the syringe was filled with 50 μM freshly prepared hemin chloride in the same buffer. The first injection of 0.5 μl, omitted from the analysis, was followed by 23 injections of 10 μl that was injected 24 times with 240 s of spacing. The titration syringe was continuously stirred at 750 rpm and at a constant temperature of 25 °C. The fitting was performed by nonlinear regression of the integrated data to a one-site binding model using ORIGIN.

The inhibition of heme binding by VHH was measured after IsdH linker-NEAT3, and the antibodies were dialyzed separately in PBS. The cell of the VP-ITC was filled with 8 μM of IsdH linker-NEAT3 supplemented with 16 μM of VHH in PBS containing 5% dimethyl sulfoxide, and the syringe was loaded with 200 μM heme in the same buffer. After the first injection of 0.5 μl (omitted from the analysis) was followed by 14 injections of 10 μl with 200 s of spacing at a constant temperature of 25 °C. The obtained data were analyzed with the program ORIGIN, and the binding of heme to linker-NEAT 3 was compared with the binding of heme to the complex of VHH with linker-NEAT3.

### Inhibition of Hb binding by VHH6

SPR experiments of VHH6 and hHb competitive binding to IsdH full-length (NEAT1–NEAT3) were performed on a Biacore T200 (GE Healthcare) by immobilizing IsdH NEAT1–NEAT3 by amine coupling (21) in a CM5 chip with acetate at pH 4.5 with an immobilization level aim of 300 RU. The competitive binding of hHb to IsdH NEAT1–NEAT3 bound to VHH was measured in a manual run by injecting 160 nM VHH6 and, immediately after, injecting 10 nM human methemoglobin. Similarly, the competitive binding of VHH6 to IsdH NEAT1–NEAT3 bound to hHb was measured in a manual run by injecting 10 nM human methemoglobin 6 and, immediately after, injecting 160 nM VHH.

### Crystallization, data collection, and refinement

Purified Isd protein (IsdH or IsdB) was mixed with VHH and subjected to SEC to purify the complex in a buffer composed of 20 mM Tris (pH 8.0) and 50 mM NaCl. The fractions were pooled together and concentrated to 10 mg/ml prior to crystallization experiments (hanging drop). For the VHH–IsdH complex, the crystallization solution was made of 0.2 M sodium chloride, 0.1 M Tris (pH 8.5), and 25% w/v PEG 3350. In the case of crystals of VHH6–IsdB, the solution contained 0.1 M Hepes (pH 7.5) and 25% w/v PEG 3350. Suitable crystals were harvested, briefly incubated in mother liquor supplemented with 20% glycerol, and transferred to liquid nitrogen for storage. Diffraction data from a single crystal were collected in beamline BL5A at the Photon Factory (Tsukuba) under cryogenic conditions (100 K). Diffraction images were processed with the program MOSFLM and merged and scaled with the program SCALA or AIMLESS (40) of the CCP4 suite (41). The structure of the protein was determined by the molecular replacement method using the coordinates of a homologous VHH (PDB entry code: 4GRW) (42) and of IsdH NEAT3 (PDB entry code: 3VTM) (16) with the program PHASER (43). The models were refined with the programs REFMAC5 (44) and built manually with COOT (45). Validation was carried out with PROCHECK (46). Data collection and refinement statistics are given in Table S3 in the Supporting information section.

### MD simulations of VHH6 in complex with IsdH linker-NEAT3

MD simulations of the VHH6 in complex with IsdH linker-NEAT3 were performed using GROMACS 2016.3 with the CHARMM36m force field and the CMAP correction (47, 48). Using the CHARMM-GUI, the structure of the complex was solvated with water in a rectangular box such that the minimum distance to the edge of the box was 15 Å under periodic boundary conditions. Sodium and chlorine ions were added to neutralize the protein charge, then further ions were added to mimic a salt solution concentration of 0.14 M. Each system was energy minimized for 5000 steps and equilibrated with NVT ensemble at 298 K for 1 ns. Further production run was performed for 280 ns with NPT ensemble (Fig. S3). A cutoff distance of 12 Å for coulomb and van der Waals interactions was used. Long-range electrostatics were evaluated through the particle mesh Ewald method (49). LINCS algorithm (50) was employed to constrain bonds involving hydrogen atoms. The time step was set to 2 fs throughout the simulations. The simulation was run three independent times for each system, and the snapshots were saved every 10 ps. Interaction energy in kcal mol^−1^ was defined as the sum of van der Waals and Coulomb interactions, which were calculated through GROMACS based on the last 50 ns of each trajectory for all the three independent replicated runs.

### Inhibition of growth assay

MRSA strains (51) USA300 (GenBank accession number: CP000255), KUH180062 (denominated MRSA49; GenBank accession number: AP020321), and KUH180129 (denominated MRSA54; GenBank accession number: AP020323) were cultured overnight in RPMI medium supplemented with 2 μM ethylenediamine-*N*, *N*′-bis (2-hydroxyphenylacetic acid) (Toronto Research Chemicals), an iron-chelating agent. The absorbance at 600 nm of overnight cultures was normalized to 1.0 and subcultured at 1/1000 dilution into RPMI supplemented with 2 μM ethylenediamine-*N*, *N*′-bis (2-hydroxyphenylacetic acid), 20 nM hHb (Sigma), and 33 nM, 66 nM, or 133 nM of antibody VHH6. Tubes were incubated at 37 °C for 17 h, and absorbance at 600 nm values was measured. In experiments where an alternative source of iron was necessary, the medium was supplemented with 0.2% THY (52), and the bacterial growth was monitored at 600 nm as outlined above.

## Data availability

The coordinates and structure factors of IsdH-NEAT3 in complex with VHH6, and of IsdB-NEAT2 in complex with VHH6 have been deposited in the PDB with entry codes 7XLD and 7XLI, respectively. All remaining data are contained within the article.

## Supporting information

This article contains supporting information.

## Conflict of interest

The authors declare that they have no conflicts of interest with the contents of this article.

## References

[1] Chambers H.F. (2001). The changing epidemiology of *Staphylococcus aureus*?. Emerg. Infect. Dis..

[2] Diekema D.J., Pfaller M.A., Schmitz F.J., Smayevsky J., Bell J., Jones R.N. (2001). Survey of infections due to Staphylococcus species: frequency of occurrence and antimicrobial susceptibility of isolates collected in the United States, Canada, Latin America, Europe, and the Western Pacific region for the SENTRY Antimicrobial Surveillance Program, 1997-1999. Clin. Infect. Dis..

[3] Mody L., Washer L.L., Kaye K.S., Gibson K., Saint S., Reyes K. (2019). Multidrug-resistant Organisms in hospitals: what is on patient hands and in their rooms?. Clin. Infect. Dis..

[4] Perkins D.R., Hall J.A., Lopez L.M., Cahuayme-Zuniga L.J. (2018). Disseminated methicillin-susceptible Staphylococcus aureus infection. J. Med. Microbiol..

[5] Sattler S. (2017). The role of the immune system beyond the fight against infection. Adv. Exp. Med. Biol..

[6] Litwin C.M., Calderwood S.B. (1993). Role of iron in regulation of virulence genes. Clin. Microbiol. Rev..

[7] Galardini M., Clermont O., Baron A., Busby B., Dion S., Schubert S. (2020). Major role of iron uptake systems in the intrinsic extra-intestinal virulence of the genus Escherichia revealed by a genome-wide association study. Plos Genet..

[8] Macdonald R., Cascio D., Collazo M.J., Phillips M., Clubb R.T. (2018). The Streptococcus pyogenes Shr protein captures human hemoglobin using two structurally unique binding domains. J. Biol. Chem..

[9] Gat O., Zaide G., Inbar I., Grosfeld H., Chitlaru T., Levy H. (2008). Characterization of Bacillus anthracis iron-regulated surface determinant (Isd) proteins containing NEAT domains. Mol. Microbiol..

[10] Zhang R., Wu R., Joachimiak G., Mazmanian S.K., Missiakas D.M., Gornicki P. (2004). Structures of sortase B from Staphylococcus aureus and Bacillus anthracis reveal catalytic amino acid triad in the active site. Structure.

[11] Dryla A., Gelbmann D., von Gabain A., Nagy E. (2003). Identification of a novel iron regulated staphylococcal surface protein with haptoglobin-haemoglobin binding activity. Mol. Microbiol..

[12] Belcher J.D., Beckman J.D., Balla G., Balla J., Vercellotti G. (2010). Heme degradation and vascular injury. Antioxid. Redox Signal..

[13] Mazmanian S.K., Skaar E.P., Gaspar A.H., Humayun M., Gornicki P., Jelenska J. (2003). Passage of heme-iron across the envelope of *Staphylococcus aureus*. Science.

[14] Pilpa R.M., Robson S.A., Villareal V.A., Wong M.L., Phillips M., Clubb R.T. (2009). Functionally distinct NEAT (NEAr transporter) domains within the *Staphylococcus aureus* IsdH/HarA protein extract heme from methemoglobin. J. Biol. Chem..

[15] Torres V.J., Pishchany G., Humayun M., Schneewind O., Skaar E.P. (2006). *Staphylococcus aureus* IsdB is a hemoglobin receptor required for heme iron utilization. J. Bacteriol..

[16] Vu N.T., Moriwaki Y., Caaveiro J.M.M., Terada T., Tsutsumi H., Hamachi I. (2013). Selective binding of antimicrobial porphyrins to the heme-receptor IsdH-NEAT3 of *Staphylococcus aureus*. Protein Sci..

[17] Watanabe M., Tanaka Y., Suenaga A., Kuroda M., Yao M., Watanabe N. (2008). Structural basis for multimeric heme complexation through a specific protein-heme interaction - the case of the third neat domain of IsdH from *Staphylococcus aureus*. J. Biol. Chem..

[18] Mazmanian S.K., Ton-That H., Su K., Schneewind O. (2002). An iron-regulated sortase anchors a class of surface protein during Staphylococcus aureus pathogenesis. Proc. Natl. Acad. Sci. U. S. A..

[19] De Genst E., Silence K., Decanniere K., Conrath K., Loris R., Kinne R. (2006). Molecular basis for the preferential cleft recognition by dromedary heavy-chain antibodies. Proc. Natl. Acad. Sci. U. S. A..

[20] Eliseev I.E., Yudenko A.N., Vysochinskaya V.V., Svirina A.A., Evstratyeva A.V., Drozhzhachih M.S. (2018). Crystal structures of a llama VHH antibody BCD090-M2 targeting human ErbB3 receptor. F1000Res.

[21] Lee C.M.Y., Iorno N., Sierro F., Christ D. (2007). Selection of human antibody fragments by phage display. Nat. Protoc..

[22] Muyldermans S. (2021). A guide to: generation and design of nanobodies. FEBS J..

[23] Vincke C., Loris R., Saerens D., Martinez-Rodriguez S., Muyldermans S., Conrath K. (2009). General strategy to humanize a camelid single-domain antibody and identification of a universal Humanized nanobody scaffold. J. Biol. Chem..

[24] Moriwaki Y., Terada T., Caaveiro J.M.M., Takaoka Y., Hamachi I., Tsumoto K. (2013). Heme binding mechanism of structurally similar iron-regulated surface determinant near transporter domains of *Staphylococcus aureus* exhibiting different affinities for heme. Biochemistry.

[25] Spirig T., Malmirchegini G.R., Zhang J., Robson S.A., Sjodt M., Liu M.Y. (2013). *Staphylococcus aureus* uses a novel multidomain receptor to break apart human hemoglobin and steal its heme. J. Biol. Chem..

[26] Valenciano-Bellido S., Caaveiro J.M.M., Morante K., Sushko T., Nakakido M., Nagatoishi S. (2022). Structure and role of the linker domain of the iron surface-determinant protein IsdH in heme transportation in Staphylococcus aureus. J. Biol. Chem..

[27] Bowden C.F.M., Chan A.C.K., Li E.J.W., Arrieta A.L., Eltis L.D., Murphy M.E.P. (2018). Structure-function analyses reveal key features in *Staphylococcus aureus* IsdB-associated unfolding of the heme-binding pocket of human hemoglobin. J. Biol. Chem..

[28] Altschul S.F., Gish W., Miller W., Myers E.W., Lipman D.J. (1990). Basic local alignment search tool. J. Mol. Biol..

[29] Grigg J.C., Ukpabi G., Gaudin C.F.M., Murphy M.E.P. (2010). Structural biology of heme binding in the *Staphylococcus aureus* Isd system. J. Inorg. Biochem..

[30] Krissinel E., Henrick K. (2007). Inference of macromolecular assemblies from crystalline state. J. Mol. Biol..

[31] Ellis-Guardiola K., Clayton J., Pham C., Mahoney B.J., Wereszczynski J., Clubb R.T. (2020). The *Staphylococcus aureus* IsdH receptor forms a dynamic complex with human hemoglobin that triggers heme release via two distinct hot spots. J. Mol. Biol..

[32] Sjodt M., Macdonald R., Marshall J.D., Clayton J., Olson J.S., Phillips M. (2018). Energetics underlying hemin extraction from human hemoglobin by *Staphylococcus aureus*. J. Biol. Chem..

[33] Bennett M.R., Bombardi R.G., Kose N., Parrish E.H., Nagel M.B., Petit R.A. (2019). Human mAbs to Staphylococcus aureus IsdA provide protection through both heme-blocking and Fc-mediated mechanisms. J. Infect. Dis..

[34] Bennett M.R., Dong J.H., Bombardi R.G., Soto C., Parrington H.M., Nargi R.S. (2019). Human V(H)1-69 gene-encoded human monoclonal antibodies against Staphylococcus aureus IsdB use at least three distinct modes of binding to inhibit bacterial growth and pathogenesis. mBio.

[35] Moriwaki Y., Caaveiro J.M.M., Tanaka Y., Tsutsumi H., Hamachi I., Tsumoto K. (2011). Molecular basis of recognition of antibacterial porphyrins by heme-transporter IsdH-NEAT3 of *Staphylococcus aureus*. Biochemistry.

[36] Maenaka K., Furuta M., Tsumoto K., Watanabe K., Ueda Y., Kumagai I. (1996). A stable phage-display system using a phagemid vector: phage display of hen egg-white lysozyme (HEL), Escherichia coli alkaline, phosphatase, and anti-HEL monoclonal antibody, HyHEL10. Biochem. Biophys. Res. Commun..

[37] Swindells M.B., Porter C.T., Couch M., Hurst J., Abhinandan K.R., Nielsen J.H. (2017). abYsis: integrated antibody sequence and structure-management, analysis, and prediction. J. Mol. Biol..

[38] Johnson G., Wu T.T. (2001). Kabat database and its applications: future directions. Nucleic Acids Res..

[39] Fischer M.J.E. (2010). Amine coupling through EDC/NHS: a practical approach. Methods Mol. Biol..

[40] Evans P. (2006). Scaling and assessment of data quality. Acta Crystallogr. D Struct. Biol..

[41] Winn M.D., Ballard C.C., Cowtan K.D., Dodson E.J., Emsley P., Evans P.R. (2011). Overview of the CCP4 suite and current developments. Acta Crystallogr. D Struct. Biol..

[42] Desmyter A., Spinelli S., Boutton C., Saunders M., Blachetot C., de Haard H. (2017). Neutralization of human interleukin 23 by multivalent nanobodies explained by the structure of cytokine-nanobody complex. Front. Immunol..

[43] Mccoy A.J., Grosse-Kunstleve R.W., Adams P.D., Winn M.D., Storoni L.C., Read R.J. (2007). Phaser crystallographic software. J. Appl. Crystallogr..

[44] Murshudov G.N., Vagin A.A., Dodson E.J. (1997). Refinement of macromolecular structures by the maximum-likelihood method. Acta Crystallogr. D Struct. Biol..

[45] Emsley P., Lohkamp B., Scott W.G., Cowtan K. (2010). Features and development of Coot. Acta Crystallogr. D Struct. Biol..

[46] Laskowski R.A., Macarthur M.W., Moss D.S., Thornton J.M. (1993). Procheck - a program to check the stereochemical quality of protein structures. J. Appl. Crystallogr..

[47] MacKerell A.D., Feig M., Brooks C.L. (2004). Improved treatment of the protein backbone in empirical force fields. J. Am. Chem. Soc..

[48] Huang J., Rauscher S., Nawrocki G., Ran T., Feig M., de Groot B.L. (2017). CHARMM36m: an improved force field for folded and intrinsically disordered proteins. Nat. Methods.

[49] Darden T., York D., Pedersen L. (1993). Particle Mesh Ewald - an N.Log(N) method for Ewald sums in large systems. J. Chem. Phys..

[50] Hess B., Bekker H., Berendsen H.J.C., Fraaije J.G.E.M. (1997). LINCS: a linear constraint solver for molecular simulations. J. Comput. Chem..

[51] Clark K., Karsch-Mizrachi I., Lipman D.J., Ostell J., Sayers E.W. (2016). GenBank. Nucleic Acids Res..

[52] Grant C.L., Pramer D. (1962). Minor element composition of yeast extract. J. Bacteriol..

[53] Pettersen E.F., Goddard T.D., Huang C.C., Couch G.S., Greenblatt D.M., Meng E.C. (2004). UCSF chimera - a visualization system for exploratory research and analysis. J. Comput. Chem..

