# Supplementary material for: Targeting hemoglobin receptors IsdH and IsdB of Staphylococcus aureus with a single VHH antibody inhibits bacterial growth
Source: J Biol Chem. 2023 Jun 15;299(9):104927. doi: 10.1016/j.jbc.2023.104927 (PMC10466926; doi:10.1016/j.jbc.2023.104927)
Supplement: Supporting Figures S1–S7 and Tables S1–S3 [file mmc1.pdf]

Supporting Information

Targeting hemoglobin receptors IsdH and IsdB of *Staphylococcus aureus* with a single VHH antibody inhibits bacterial growth.

Sandra Valenciano-Bellido[1], Jose M. M. Caaveiro[2, *], Makoto Nakakido[1,3], Daisuke Kuroda[1,4], Chihiro Aikawa[5], Ichiro Nakagawa[5], and Kouhei Tsumoto[1,3,6,*]

[1]*Department of Bioengineering, School of Engineering, The University of Tokyo, 7-3-1 Hongo, Bunkyo-ku, Tokyo 113-8656, Japan;* [2]*Laboratory of Global Healthcare, Graduate School of Pharmaceutical Sciences, Kyushu University, 3-1-1 Maidashi, Higashi-ku, Fukuoka 812-8582, Japan;* [3]*Department of Chemistry and Biotechnology, Graduate School of Engineering, The University of Tokyo, 7-3-1 Hongo, Bunkyo-ku, Tokyo 113-8656, Japan;* [4]*Research Center for Drug and Vaccine Development, National Institute of Infectious Diseases, Tokyo 162-8640, Japan;* [5]*Department of Microbiology, Graduate School of Medicine, Kyoto University, Yoshida-Konoe-cho, Sakyo-ku, Kyoto 606-8501, Japan;* [6]*Institute of Medical Science, The University of Tokyo, 4-6-1 Shirokanedai, Minato-ku, Tokyo 108-8639, Japan.*

* Corresponding author: Jose M.M. Caaveiro (jose@phar.kyushu-u.ac.jp)

* Corresponding author: Kouhei Tsumoto (tsumoto@bioeng.t.u-tokyo.ac.jp)

SUPPLEMENTARY TABLES

Table S1. Sequences of antibodies selected in ELISA. Residues in red are not conserved.

VHH N°	FR1	CDR1	FR2	CDR2
	1 … 20		40	60
2	**EL**QLVESGGGLVQPGGSLSLSCEVS	GFSFDDVDNFIIA	WFRQAPGKEREGVS	FLRKYDMSTYYAESVKG
6	**EL**QLVESGGGLVQPGGSLSLSCEVS	GFSFDDVDNFIIA	WFRQAPGKEREGVS	FLRKYDMSTYYAESVKG
29	**EV**QLVESGGGLVQPGGSLSLSCEVS	GFSFDDVDNFIIA	WFRQAPGKEREGVS	FLRKYDMSTYYAESVKG
32	**EV**QLVESGGGLVQPGGSLSLSCEVS	GFSFDDVDNFIIA	WFRQAPGKEREGVS	FLRKYDMSTYYAESVKG
35	**QL**QLVESGGGLVQPGGSLSLSCEVS	GFSFDDVDNFIIA	WFRQAPGKEREGVS	FLRKYDMSTYYAESVKG

VHH N°	FR3	CDR3	FR4
	80 … 100		120 … 125
2	RFTISSDNARDTVYLQMTNLKPEDTAVYYCAL	DREGFVFEQGMDF	WGKGTQVTVSS
6	RFTISSDNARDTVYLQMTNLKPEDTAVYYCAL	DREGFVFEQGMDF	WGKGTQVTVSS
29	RFTISSDNARDTVYLQMTNLKPEDTAVYYCAL	DREGFVFEQGMDF	WGKGTQVTVSS
32	RFTISSDNARDTVYLQMTNLKPEDTAVYYCAL	DREGFVFEQGMDF	WGKGTQVTVSS
35	RFTISSDNARDTVYLQMTNLKPEDTAVYYCAL	DREGFVFEQGMDF	WGKGTQVTVSS

Table S2. List of primers employed for the preparation of alanine mutants of VHH6.

VHH6 mutant	Forward Primer (5´- 3´)	Reverse Primer (5´- 3´)
D33A	GTTTCGATGATGTCGCTAATTTCATCATAGCCTGG	CCAGGCTATGATGAAATTAGCGACATCATCGAAAC
R55A	GCGAAGTATGATATGAGTACATACTATGCGGAGTCCGTGAAGG	GAGAAATGAGACCCCTTCACGCTCTTTCCC
Y57A	CTCATTTCTCAGGAAGGCTGATATGAGTACATACTATG	CATAGTATGTACTCATATCAGCCTTCCTGAGAAATGAG
Y62A	GCGTATGCGGAGTCCGTGAAGGGCC	TGTACTCATATCATACTTCCTGAGAAATGAGACCCCTTC
E104A	GCGGGCTTTGTTTTTGAGCAGGGCATGGAC	CCTATCTAGTGCACAGTAATATACGGCCGTGTCTTC
F106A	GTGCACTAGATAGGGAGGGCGCTGT	ACAGCGCCCTCCCTATCTAGTGCAC
V107A	GCGTTTGAGCAGGGCATGGACTTCTGGG	AAAGCCCTCCCTATCTAGTGCACAGTAATATACGG
F108A	GCGGAGCAGGGCATGGACTTCTGGGG	AACCGCGCCCTCCCTATCTAGTGCACAGTAATATACGG

Table S3: Data collection and refinement statistics.
Statistical values given in parenthesis refer to the highest resolution bin.

**Data Collection**	**IsdH-NEAT3 + VHH6**	**IsdB-NEAT2 + VHH6**
Space Group	P 1 2_1 1	P 2_1 2_1 2_1
Unit cell		
a, b, c (Å)	53.1, 49.4, 53.9	40.3, 48.6, 140.7
α, β, γ (°)	90.0, 99.5, 90.0	90.0, 90.0, 90.0
Resolution (Å)	40.9 - 1.65 (1.74 - 1.65)	40.0 - 1.70 (1.79 - 1.70)
Wavelength	1.0000	1.0000
Observations	149,228 (20,335)	262,412 (31,711)
Unique reflections	32,081 (4,588)	31,303 (4,429)
$R_{merge.}$	0.068 (0.642)	0.068 (0.828)
$R_{p.i.m.}$	0.035 (0.333)	0.025 (0.325)
$CC_{1/2}$	0.999 (0.758)	0.998 (0.827)
$I / \sigma (I)$	13.4 (2.1)	14.7 (2.3)
Multiplicity	4.7 (4.4)	8.4 (7.2)
Completeness (%)	96.1 (94.9)	99.8 (98.7)
**Refinement Statistics**		
Resolution (Å)	40.9 - 1.65 (1.74 – 1.65)	40.0 - 1.70 (1.79 - 1.70)
R_{work} / R_{free} (%)	14.8 / 19.7	20.9 / 23.7
No. protein chains	2	2
No. atoms		
VHH	995	992
NEAT	908	914
Other (no solvent)	2	2
Water	200	111
B-factor (Å^2)		
VHH	25.8	33.5
NEAT	21.0	59.1
Other (no solvent)	24.6	46.7
Water	31.4	38.9
Ramachandran Plot		
Preferred (%)	88.9	88.9
Allowed (%)	11.1	11.1
Outliers (%)	0	0
RMSD Bond (Å)	0.008	0.014
RMSD Angle (°)	1.51	1.78
PDB entry code	7XLD	7XLI

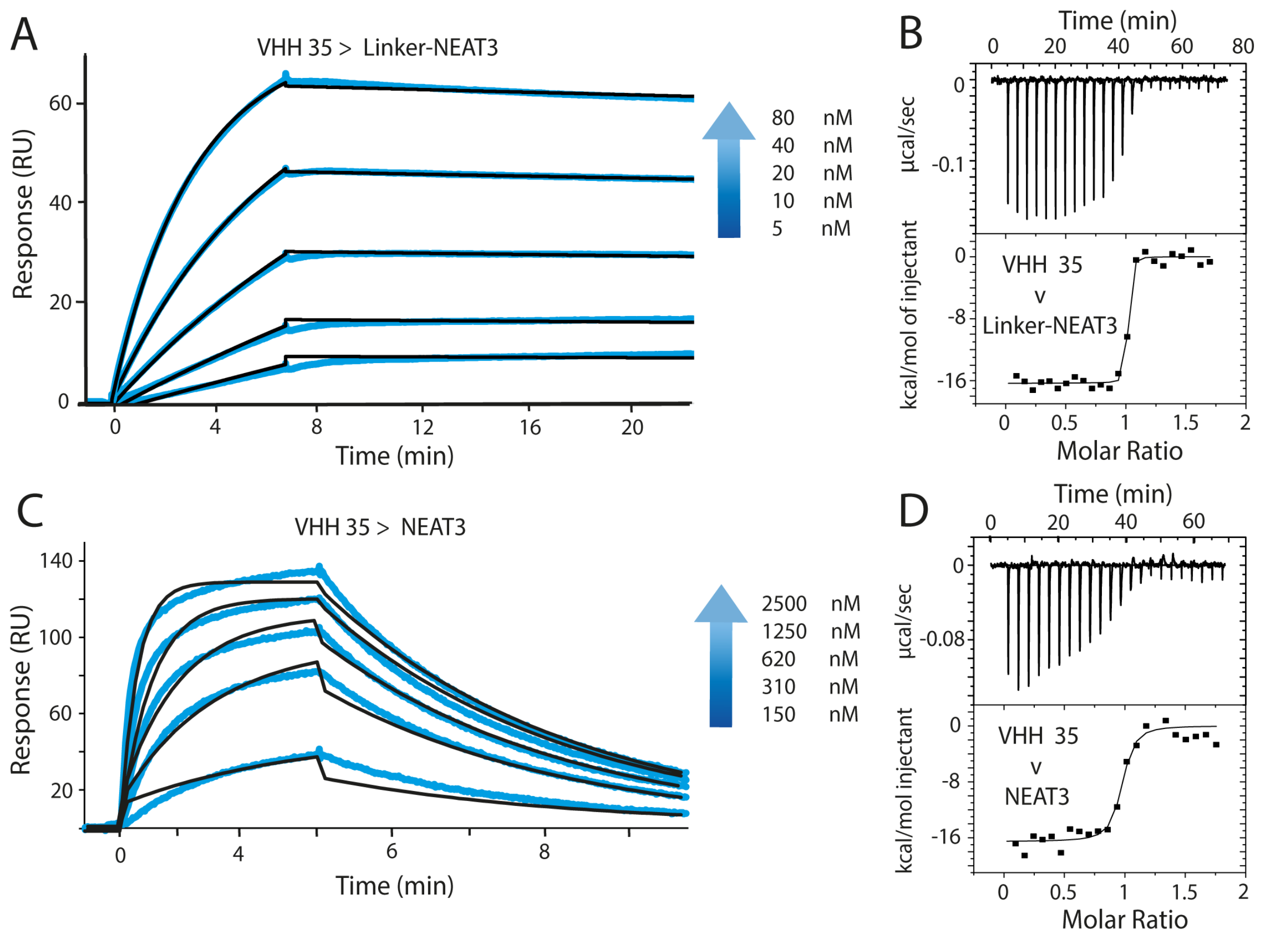

Figure S1: **Binding of VHH35 to IsdH.** Binding of VHH35 to IsdH linker-NEAT3 examined by **(a)** SPR and **(b)** ITC. Similarly, binding of VHH35 to IsdH NEAT3 examined by **(c)** SPR and **(d)** ITC. The results shown are representative of independent duplicates. The ITC and SPR experiments were performed and analyzed as described in materials and methods and the corresponding parameters are given in Table 1.

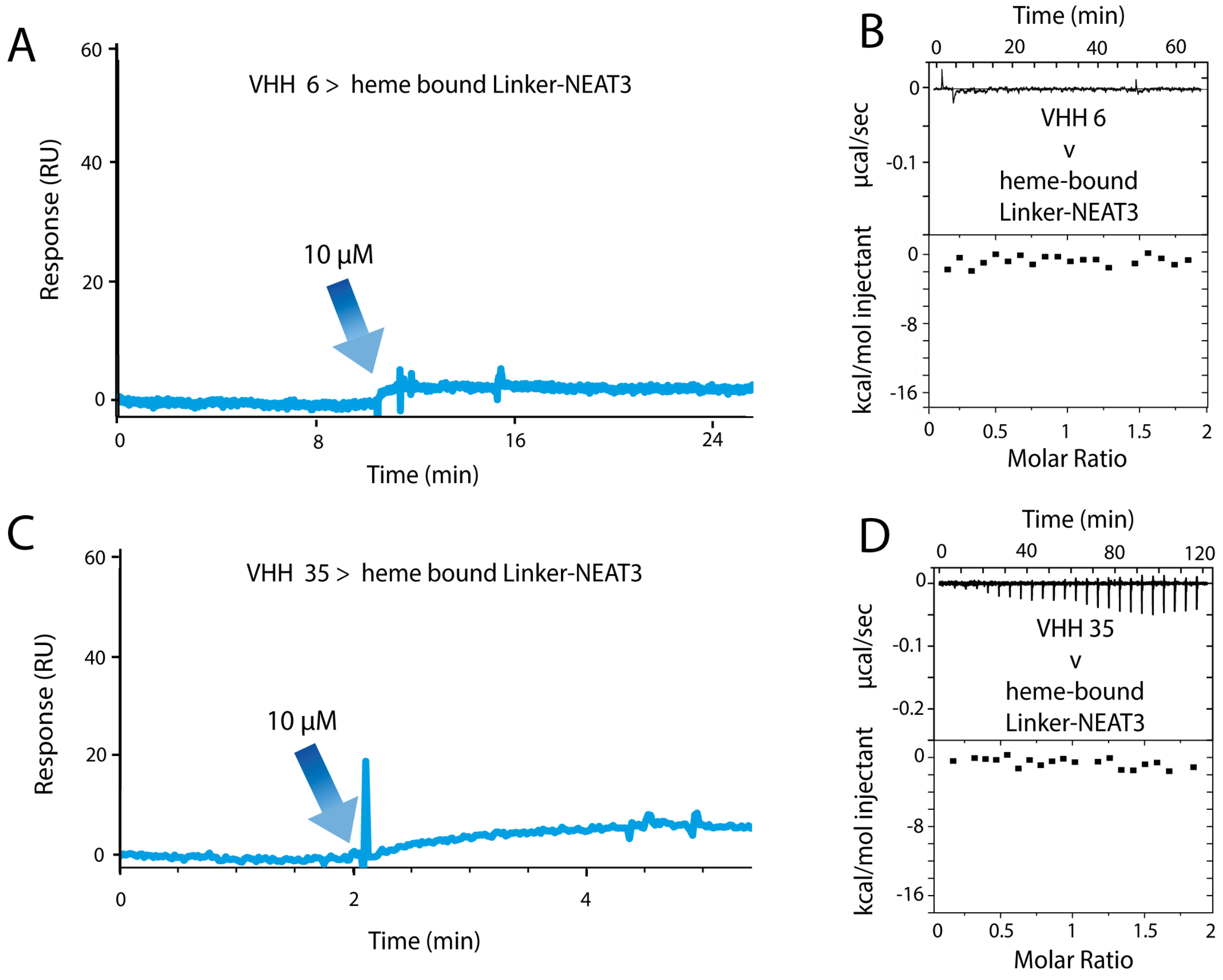

Figure S2. Pre-incubation of IsdH linker-NEAT3 with heme prevents binding of VHHs. The possible binding of VHH6 to the complex IsdH linker-NEAT3 with heme was evaluated by **(a)** SPR and **(b)** ITC. Analogous experiment, but using VHH35, evaluated by **(c)** SPR and **(d)** ITC. The experiments were performed and analyzed as described in Materials and methods.

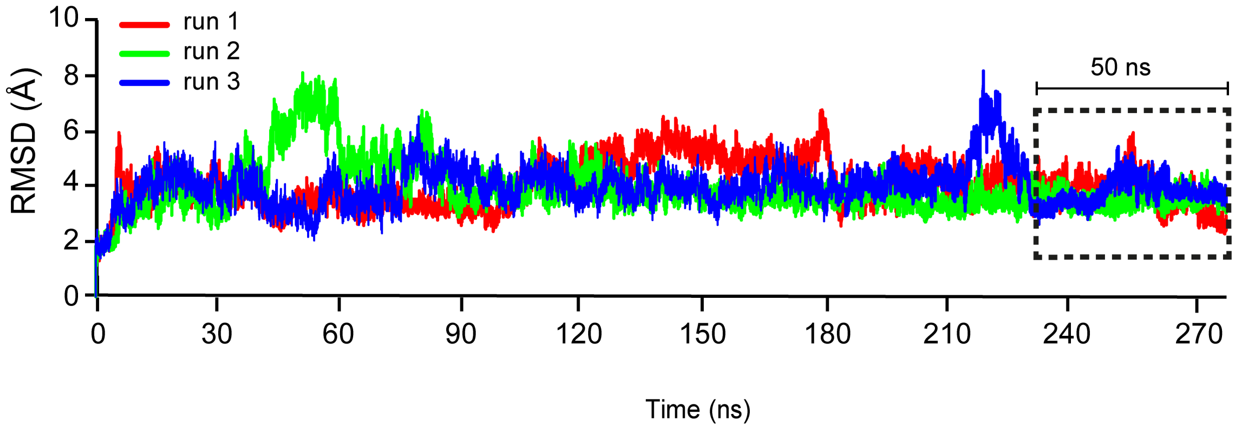

Figure S3: MD simulations. RMSD of α carbons of the complex between VHH6 and IsdH linker-NEAT3 in three different trajectories for a total of 280 ns. The square (dashed line) indicates the last 50 ns selected for analysis of Figure 6 of the main text.

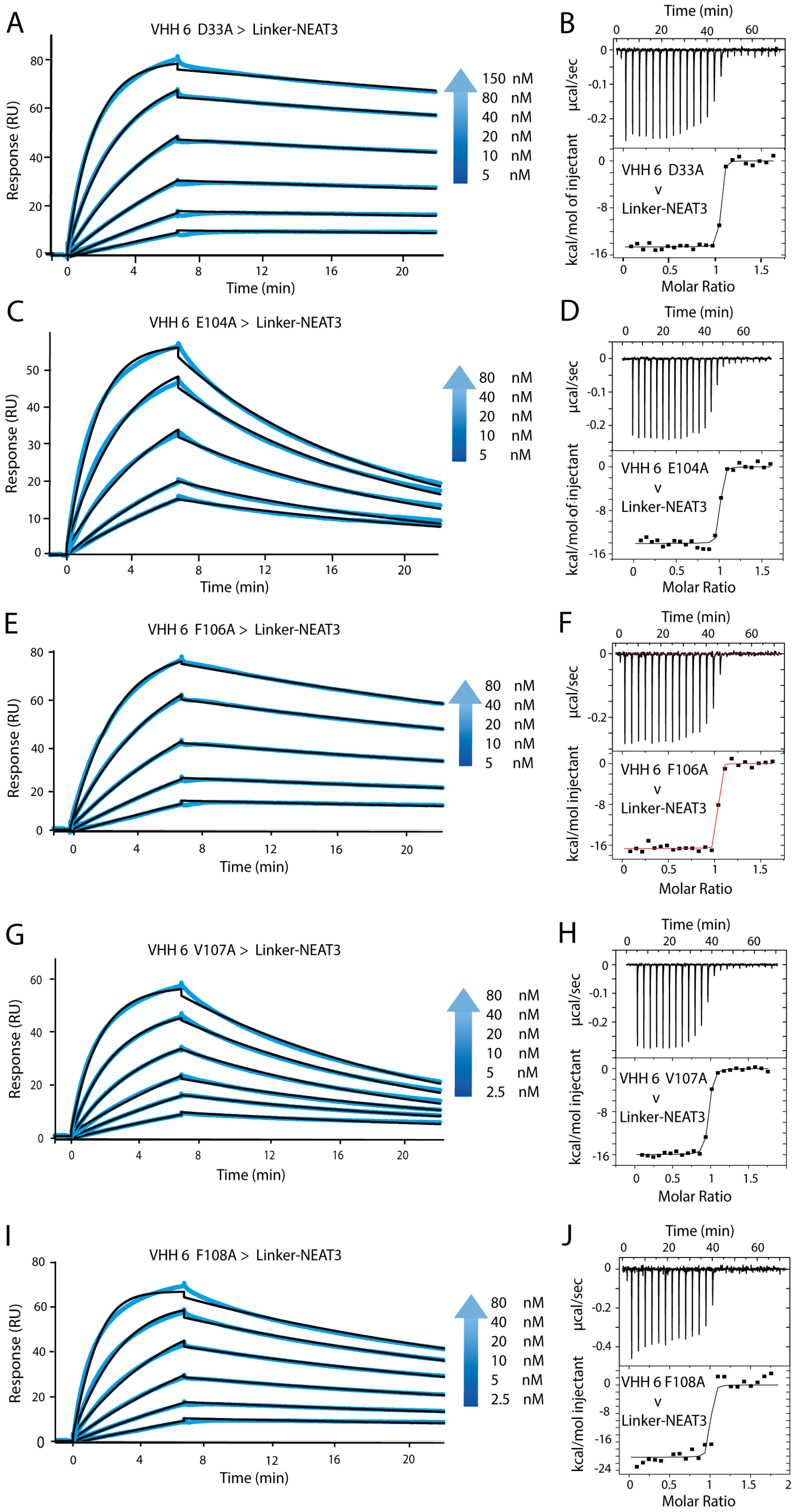
A
VHH 6 D33A > Linker-NEAT3
Response (RU)
Time (min)
150 nM
80 nM
40 nM
20 nM
10 nM
5 nM
B
Time (min)
µcal/sec
kcal/mol of injectant
VHH 6 D33A
v
Linker-NEAT3
Molar Ratio
C
VHH 6 E104A > Linker-NEAT3
Response (RU)
Time (min)
80 nM
40 nM
20 nM
10 nM
5 nM
D
Time (min)
µcal/sec
kcal/mol of injectant
VHH 6 E104A
v
Linker-NEAT3
Molar Ratio
E
VHH 6 F106A > Linker-NEAT3
Response (RU)
Time (min)
80 nM
40 nM
20 nM
10 nM
5 nM
F
Time (min)
µcal/sec
kcal/mol injectant
VHH 6 F106A
v
Linker-NEAT3
Molar Ratio
G
VHH 6 V107A > Linker-NEAT3
Response (RU)
Time (min)
80 nM
40 nM
20 nM
10 nM
5 nM
2.5 nM
H
Time (min)
µcal/sec
kcal/mol injectant
VHH 6 V107A
v
Linker-NEAT3
Molar Ratio
I
VHH 6 F108A > Linker-NEAT3
Response (RU)
Time (min)
80 nM
40 nM
20 nM
10 nM
5 nM
2.5 nM
J
Time (min)
µcal/sec
kcal/mol injectant
VHH 6 F108A
v
Linker-NEAT3
Molar Ratio

(from the previous page)

Figure S4: **Binding of single alanine mutants of VHH6 to IsdH linker-NEAT3.** The left and right panels correspond to SPR and ITC data, respectively. The following mutants of VHH were examined: **(a, b)** D33A, **(c, d)** E104A, **(e, f)** F106A, **(g, h)** V107A, and **(i, j)** F108A. The sensorgrams and binding isotherms shown in the figure are representative of independent duplicates. The ITC and SPR experiments were performed and analyzed as described in materials and methods and the kinetic and thermodynamic parameters determined are given in Table 2.

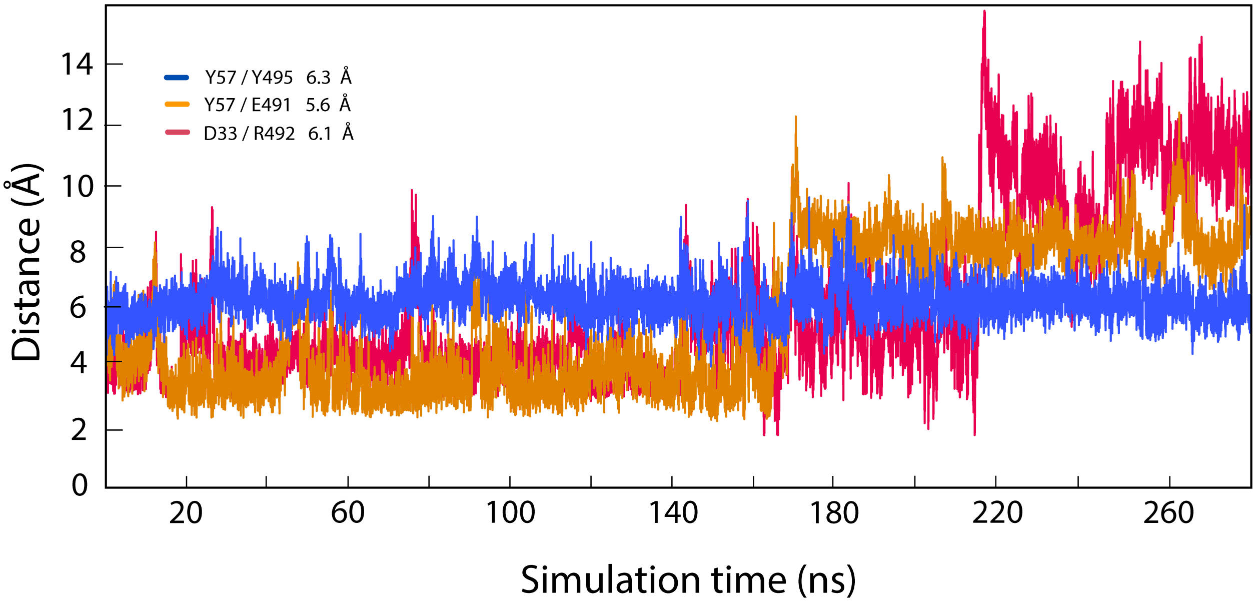

Figure S5. Distances between selected residues of VHH6 and IsdH linker-NEAT3 (MD simulations). Distances (in Å) between key residues of VHH6 and IsdH linker-NEAT3 calculated from 280 ns of MD simulations. The average distances are indicated in the panel. The distances were calculated using UCSF Chimera from the trajectory generated in GROMACS 2016.335, using the CHARMM36m force field within the CHARMM-GUI.

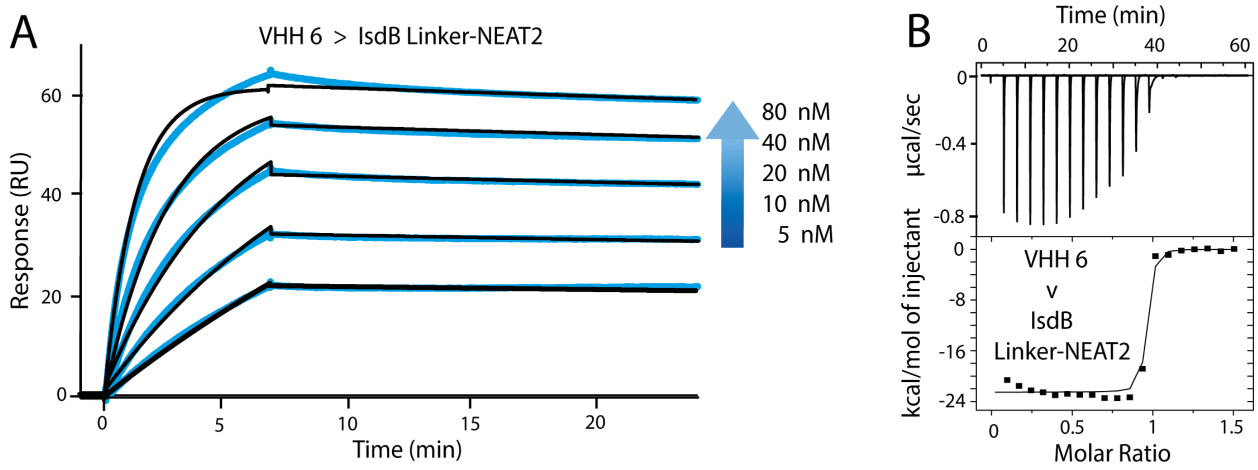

Figure S6: Binding of VHH6 to IsdB linker-NEAT2. (a) SPR. **(b)** ITC binding. The data shown are representative of independent duplicates. The experiments were performed and analyzed as described in materials and methods. The corresponding parameters are given in Table 3.

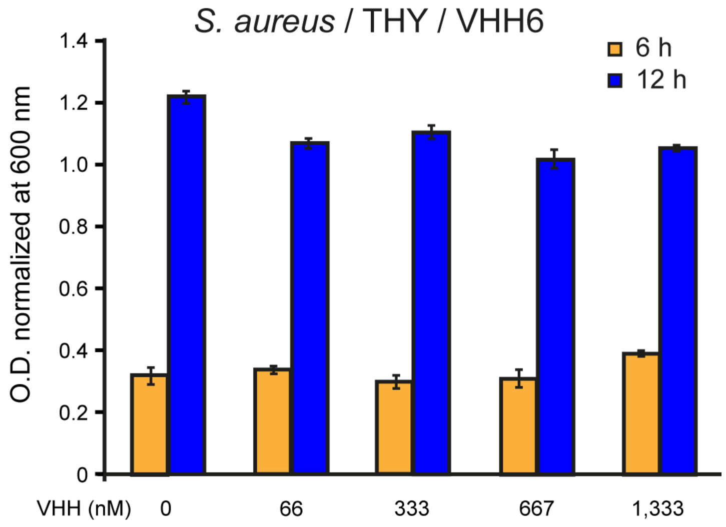

Figure S7. *S. aureus* growth inhibition assay employing alternative sources of iron. Growth of *S. aureus* in medium supplemented with THY medium containing alternative sources of iron different to that of hemoglobin. Estimated final concentration of elemental iron was greater than 1 µM. For each assay the optical density (O.D.) at 600 nm was measured and normalized to 1.0 after six and after twelve hours, in yellow and blue, respectively. The standard deviation errors are indicated with black lines.
